# Identifying Novel Inhibitors for Hepatic Organic Anion Transporting Polypeptides by Machine Learning-Based Virtual Screening

**DOI:** 10.1021/acs.jcim.1c01460

**Published:** 2022-03-11

**Authors:** Alzbeta Tuerkova, Brandon J. Bongers, Ulf Norinder, Orsolya Ungvári, Virág Székely, Andrey Tarnovskiy, Gergely Szakács, Csilla Özvegy-Laczka, Gerard J. P. van Westen, Barbara Zdrazil

**Affiliations:** † Department of Pharmaceutical Sciences, Division of Pharmaceutical Chemistry, University of Vienna, Althanstraße 14, A-1090 Vienna, Austria; ‡ Division of Drug Discovery and Safety, Leiden Academic Centre for Drug Research, Leiden University, P.O. Box 9502, 2300 RA Leiden, The Netherlands; § Department of Pharmaceutical Biosciences, Uppsala University, Box 591, SE-75124 Uppsala, Sweden; ∥ MTM Research Centre, School of Science and Technology, Örebro University, SE-70182 Örebro, Sweden; ⊥ Drug Resistance Research Group, Institute of Enzymology, RCNS, Eötvös Loránd Research Network, Magyar tudósok krt. 2, H-1117 Budapest, Hungary; # Doctoral School of Biology and Institute of Biology, ELTE Eötvös Loránd University, Pázmány P. stny. 1/C, H-1117 Budapest, Hungary; ∇ Enamine Ltd., 78 Chervonotkatska Street, 02094 Kyiv, Ukraine; ○ Department of Medicine I, Institute of Cancer Research, Comprehensive Cancer Center, Medical University of Vienna, A-1090 Vienna, Austria

## Abstract

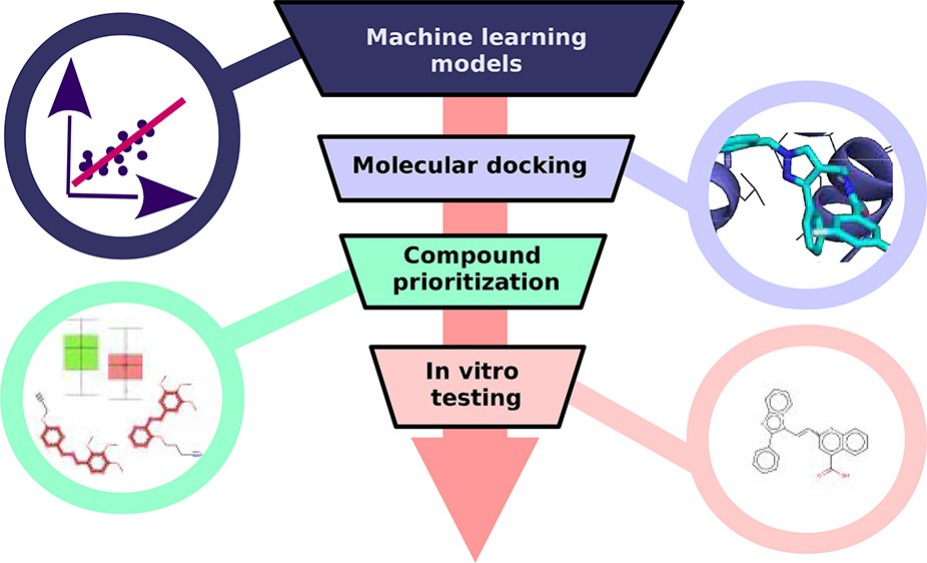

Integration of statistical learning methods with structure-based modeling approaches is a contemporary strategy to identify novel lead compounds in drug discovery. Hepatic organic anion transporting polypeptides (OATP1B1, OATP1B3, and OATP2B1) are classical off-targets, and it is well recognized that their ability to interfere with a wide range of chemically unrelated drugs, environmental chemicals, or food additives can lead to unwanted adverse effects like liver toxicity and drug–drug or drug–food interactions. Therefore, the identification of novel (tool) compounds for hepatic OATPs by virtual screening approaches and subsequent experimental validation is a major asset for elucidating structure–function relationships of (related) transporters: they enhance our understanding about molecular determinants and structural aspects of hepatic OATPs driving ligand binding and selectivity. In the present study, we performed a consensus virtual screening approach by using different types of machine learning models (proteochemometric models, conformal prediction models, and XGBoost models for hepatic OATPs), followed by molecular docking of preselected hits using previously established structural models for hepatic OATPs. Screening the diverse *REAL* drug-like set (Enamine) shows a comparable hit rate for OATP1B1 (36% actives) and OATP1B3 (32% actives), while the hit rate for OATP2B1 was even higher (66% actives). Percentage inhibition values for 44 selected compounds were determined using dedicated *in vitro* assays and guided the prioritization of several highly potent novel hepatic OATP inhibitors: six (strong) OATP2B1 inhibitors (IC_50_ values ranging from 0.04 to 6 μM), three OATP1B1 inhibitors (2.69 to 10 μM), and five OATP1B3 inhibitors (1.53 to 10 μM) were identified. Strikingly, two novel OATP2B1 inhibitors were uncovered (**C7** and **H5**) which show high affinity (IC_50_ values: 40 nM and 390 nM) comparable to the recently described estrone-based inhibitor (IC_50_ = 41 nM). A molecularly detailed explanation for the observed differences in ligand binding to the three transporters is given by means of structural comparison of the detected binding sites and docking poses.

## Introduction

The organic anion transporting polypeptides OATP1B1, OATP1B3, and OATP2B1 are commonly expressed at the basolateral membrane of hepatocytes. They are involved in the hepatobiliary transport of various compounds, such as bile salts, bilirubin, hormones (and their conjugated forms), nutrients, and xenobiotics (including many drugs). Hepatic OATPs exhibit broad substrate specificity with partially overlapping substrate/inhibitor profiles. Several specific compounds for OATP1B1 (e.g., pravastatin),^1^ OATP1B3 (e.g., cholecystokinin octapeptide),^2^ and OATP2B1 (e.g., erlotinib)^1^ have been identified. Similar ligand profiles across the three transporters might be attributed to the degree of their sequence similarities; while OATP1B1 and OATP1B3 share about 80% sequence identity, OATP2B1 is phylogenetically more distant (∼30% sequence identity with the OATP1B subfamily, Supporting Information Table S1).

Given their high expression levels at the sinusoidal membrane of hepatocytes, OATPs are being increasingly recognized for their contribution to normal liver function, such as enterohepatic circulation of bile salts or metabolism of bilirubin.^3,4^ Defects in the expression and function of these transporters might affect proper liver physiology, which can result in manifold clinical consequences. For example, impaired uptake of bilirubin leads to elevated concentration of bilirubin in the blood, which, in turn, can result in the manifestation of Rotor syndrome.^5^ Rotor syndrome is a rare, conjugated hyperbilirubinemia, induced by simultaneous mutations in SLCO1B1 and SLCO1B3 genes.

OATP-mediated drug–drug interactions are another reason why all three hepatic OATP transporters are listed among the clinically relevant transporters in the White Paper by the International Transporter Consortium (ITC) and also by the U.S. Food and Drug Administration.^6^ However, little is known about their structural aspects of ligand recognition and selectivity (especially in the case of OATP2B1). Examining novel therapeutic candidates for their possible interaction with hepatic OATPs is a recommended safety assessment strategy in the early phase of drug discovery. In addition, having new (selective) ligands to be used as tool compounds would help to further elucidate the biological role of these transporters.

Integrating artificial intelligence with structure-based approaches into a single virtual screening pipeline is a promising strategy to detect novel compounds in a more efficient (and therefore less cost-intensive) manner than the sole use of simpler ligand-based approaches (such as simple QSAR models) or the sole use of docking approaches.^7^ A very good overview of the different flavors of ML-based virtual screening approaches successfully employed by other researchers is given in a recent review by Kimber et al.^8^

The most comprehensive screening study for hepatic OATPs done so far was performed by Karlgren et al.^1^ In that study, 225 drug-like compounds were tested for their activity on OATP1B1, OATP1B3, and OATP2B1. Out of these, 91 OATP inhibitors with different or overlapping profiles across the three hepatic OATPs were identified. Among those, some specific OATP1B1 (pravastatin, IC_50_ = 3.6 μM) and OATP2B1 (erlotinib, IC_50_ = 0.55 μM) inhibitors were found. The authors combined *in vitro* (i.e., single point inhibition experiments, IC_50_ values determination, *in vitro* to *in vivo* extrapolations using the maximal transport activity) and *in silico* (i.e., binary classification, *in vivo* uptake clearance prediction) models to perform such an extensive screening study.

In another screening study, several OATP1B1 inhibitors with *K*_i_ values ranging from 0.06 to 6.5 μM were identified, and proteochemometric models were subsequently developed utilizing *in vitro* data.^9^ The authors subsequently performed prospective validation using a Random Forest model.

Finally, Khuri et al. identified novel OATP2B1 inhibitors by applying a combination of Random Forest modeling and structure-based virtual screening (VS).^10^ At the first stage, a Random Forest model was used to screen DrugBank.^11^ Then, multiple comparative structural models corresponding to distinct transporter conformational states were subjected to docking calculations, which led to the prioritization of 33 putative OATP2B1 inhibitors. Of these, three compounds were confirmed as OATP2B1 inhibitors.

In our recent study, we explored potential binding modes of steroid-like compounds in the three hepatic OATPs by means of a rigorous computational pipeline combining exhaustive sampling of protein template conformations by using elastic network models, model generation on the basis of multiple conformers, ensemble docking, and prioritization of the final models on the basis of ligand enrichment. Our computational and experimental strategy to validate the findings has proven successful in delivering meaningful explanations for efficacy and selectivity of a set of known (publicly available) and novel (in-house synthesized and experimentally tested) steroidal inhibitors of OAP1B1, OATP1B3, and OATP2B1.^12^

In the present study, we made use of the already established and successfully deployed structural models for using them in a predictive fashion. For this purpose, a data set of inhibitors and substrates of the three hepatic OATPs collected from the public domain and published earlier by our group^13^ served in order to train a set of machine learning (ML) models including different techniques: proteochemometric (PCM) models, conformal prediction (CP) models, and XGBoost models. These models were subsequently used to screen the diverse *REAL* drug-like set (a subset of ENAMINE *REAL* with 21M compounds).^26^ In a consecutive step, the previously established structural models for the three hepatic OATPs have been used to prioritize hits from the ML-based screening.

Here, we show that a consensus virtual screening approach was very successful with hit rates of 36%, 32%, or 66% in the case of OATP1B1, OATP1B3, or OATP2B1, respectively. Measurements (percentage inhibition) for a data set of 44 novel compounds were determined and guided the selection of the most promising compounds for IC_50_ determination. For six strong OATP inhibitors, binding mode hypotheses have been studied in more detail delivering insights into molecularly detailed explanations for ligand affinity and selectivity. Structural comparison of the detected binding sites across the three transporters unraveled remarkable differences in the localization of aromatic residues in OATP1B1/OATP1B3 vs OATP2B1 delivering a potential explanation for ligand selectivity. In summary, the machine learning-based virtual screening approach identified 29 novel OATP inhibitors.

## Materials and Methods

All data, code, workflows, and models used or created in this study are available from an open GitHub repository: https://github.com/AlzbetaTuerkova/VirtualScreening.

**Figure 1:**
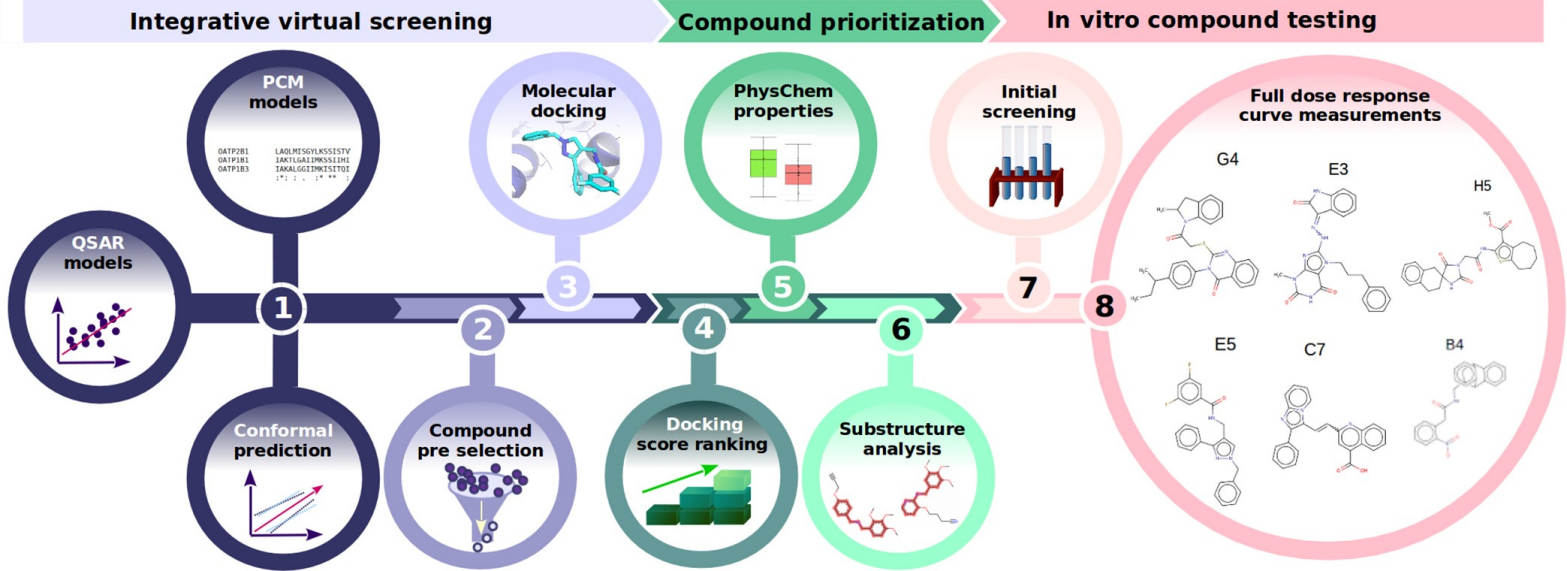
Schematic overview of the integrative ML-based VS strategy, compound prioritization, and experimental testing of the selected hits.

An overview of the employed VS pipeline is visualized in Figure 1.

### Data Sets Used

Data sets for OATP1B1, OATP1B3, and OATP2B1 substrates/nonsubstrates and inhibitors/noninhibitors were previously retrieved from five different data sources (ChEMBL, UCSF-FDA TransPortal, DrugBank, Metrabase, IUPHAR).^13^ The number of enumerated compounds per transporter is listed in Table 1. Supporting Information Figure S1 shows the number of active compounds for the respective transporters as well as numbers of compounds with multitarget activity. Classification into active and inactive measurements was achieved by setting a cutoff at 10 μM (anything below the cutoff is annotated as “active”) and considering different bioactivity end points (*K*_i_, IC_50_, EC_50_, *K*_m_, percentage inhibition). OATP data sets were standardized via the Atkinson standardization protocol (available at https://github.com/flatkinson/standardiser).

**Table 1:** Numbers of Unique Compounds per Transporter Used in This Study^a^

activity class	OATP1B1	OATP1B3	OATP2B1
actives	360	225	78
inactives	1017	1063	171
total number	1377	1288	249
actives/inactives	1:2.8	1:4.7	1:2.2

a Compounds might appear annotated to more than one target.

### Multiple Sequence Alignment

In order to prepare the protein sequences for PCM modeling, multiple sequence alignment of all human OATPs was performed using the PROMALS3D server (available at http://prodata.swmed.edu/promals3d/promals3d.php).^14^ Further, the PSIPRED tool (available at http://bioinf.cs.ucl.ac.uk/psipred/) was used to predict secondary structures of hepatic OATPs.^15^ In order to identify putative transmembrane helices of OATPs, the OCTOPUS tool (available at http://octopus.cbr.su.se/index.php) served to predict their membrane topology.^16^ The generated multiple sequence alignment with highlighted transmembrane regions is provided as additional information on GitHub.

### Conformal Prediction Models

Conformal Prediction (CP) is a framework for deriving machine learning models, e.g., QSAR models, at a predefined level of significance, i.e., error rate.^17^ A conformal predictor will make *valid* predictions on new test compounds corresponding to the user-defined significance level provided that the data is *exchangeable*. In a binary classification problem, a set of class labels is assigned to new compounds by comparing them to calibration set classifications with known classes (active and inactive).

A new compound is assigned a class label if the prediction outcome for the compound is higher than the set significance level, i.e., similar enough to the corresponding predictions for the calibration set compounds for the two classes A (active) and I (inactive), respectively. Thus, for a binary classification problem, there are four possible outcomes. A new compound can be assigned to either of the two classes, assigned to both classes (*both* classification) or none of the classes (*empty* classification). Compounds assigned to the *empty* class are considered out-of-domain of the model for which reliable prediction cannot be produced. This includes taking the applicability domain into account as part of the framework.^18^

To assess the similarity between the new compound and the respective calibration set compounds for each class, a similarity (conformity) measure must be defined. In this work, the percentage of trees in the Random Forest ensemble predicting each of the two classes (class probability) is used as that measure.

The assignment to a class is then performed by comparing the class probability against the corresponding sorted list of class probabilities for the calibration set (in descending order) associated with each Random Forest (RF) model. The predicted class probabilities for classes A and I of the new compound are placed in the sorted list of calibration set probabilities for the respective classes A and I thus adding one entry to the list for each class. The position of the new compound in each of these two sorted lists is determined, and the fraction of calibration set compounds with lower probabilities is calculated. This fraction is then compared to the significance level set by the user. For a new compound to be assigned a class, the calculated fraction must be larger or equal to the set significance level.

*Validity* and *efficiency* are two measures that indicate the performance of a conformal predictor. The predictor is *valid* if the percentage of errors does not exceed the set significance level. This is actually taken for granted since *exchangeability* of the data set is assumed. In conformal prediction, a prediction is considered correct if it includes the correct predicted class label, which means that *both* predictions are always correct, and *vice versa*, *empty* predictions are never correct (i.e., always erroneous). The conformal prediction *efficiency* is calculated as the percentage of the total number of single class predictions, regardless of whether they are correct or not, in relation to the total number of predicted compounds. *Validity* and *efficiency* are calculated for each of the two classes. Using two calibration sets, one for each class, CP guarantees *validity* for both classes. This form of CP is referred to as Mondrian CP.

We have used the RF algorithm^19^ for deriving the underlying models in our conformal predictors. The models were developed using Python, Scikit-learn^20^ version 0.20.4, and the nonconformist package^21^ version 1.2.5. Binary classification models were built based on RF using the Scikit-learn RandomForestClassifier with 100 trees and all other options set at the default value.

#### Model Development

The available data sets were randomly divided into a *proper* training set (70%) and calibration set (20%). The RF model was derived using the *proper* training set and the calibration set used for predicting the conformal prediction *p*-values of the new compounds (test sets).

Twenty pairs of *proper* and calibration sets were generated and used to predict the test sets (Aggregated CP).^22^ This produced 20 CP *p*-values for each class and each predicted compound. The median *p*-value for each class of each predicted compound was then used to determine the final class assignment.

### Proteochemometric and QSAR Models

For the Proteochemometrics (PCM) and RF models, the data was trained on the complete data set. For these compounds, the following properties were calculated: AlogP, molecular weight, number of H donors and acceptors, rotatable bonds, number of atoms, rings, aromatic rings and fragments, NPlusO count, molecular solubility, surface area, polar surface area, and polar SASA. In addition, functional extended connectivity fingerprints were added (FCFP_6) to define the compounds more precisely.^23^ This data was fed into a gradient boosting method named XGBoost in the Pipeline Pilot 2018 suite, with the following settings: max trees: 100, learning rate: 0.3, max depth: 7, data fraction: 1.0, descriptor fraction: 0.7, gbtree as booster function and seed: 12345. For the PCM models, additional protein descriptors were calculated using Z-scales^24^ using the first three Z-scales per amino acid.^25^

### Combined Models

The created models were separated into several categories. First, for both CP and XGBoost predictions, models were created that either include or did not include protein descriptors (PCM models vs QSAR models). Subsequently, models were created that predicted activity on OATP1B1, OATP1B3, and OATP2B1 together (thereafter called “general models”) for both the PCM and QSAR types. Then, predictions were made on single targets, whereby new models were trained for each of the OATPs. For the XGBoost models, this meant that a consolidated model was formed with the three individual models on the QSAR side, while having the single PCM model predict only one “active” activity. This resulted in 16 separate prediction sets (eight from CP and eight from XGBoost modeling, where each method had four PCM prediction sets and four QSAR prediction sets).

Performances of the models were estimated by performing internal 5-fold cross-validation (CV).

### Machine Learning-Based Prescreening

A data set of untested compounds was constructed to test the aforementioned ML models for potential hits. Data was collected from the diverse *REAL* drug-like set (Enamine),^26^ and of this set, the virtual HTS collection was chosen and provided with calculated FCFP_6 fingerprints, resulting in a data set of 1,963,425 compounds. The predicted values for all compounds were ranked by affinity for each model type and deemed active on any of the OATP proteins if this value was higher than 6.5 log units (approximately 300 nm). From this ranking, we selected the top 250 predicted actives from each of the 16 models (where possible) and combined them (by duplicate removal) to a list of 3,291 compounds that were used in the subsequent structure-based VS step.

### Structure-Based Virtual Screening

#### Comparative Modeling

Recently, we constructed comparative protein models of OATP1B1, OATP1B3, and OATP2B1.^12^ Briefly, leveraging fold-recognition methods, a suitable template was detected (fucose transporter in an outward open conformation, PDB ID: 3O7Q). Elastic network models calculated for the template structure via normal mode analysis served to sample protein conformational space. Finally, ensemble docking into multiple conformations helped to identify the most suitable structure for VS of hepatic OATPs.

Comparison of the central binding site found in the three OATP transporters was done by calculating volumetric maps by using the POVME plugin (version 3.0) in PyMol.^27^

#### Molecular Docking of ML-Based Preselected Hits

Potential interaction sites in OATP1B1, OATP1B3, and OATP2B1 transporter structures were mapped via the small molecule mapping server FTMap (available at https://ftmap.bu.edu/serverhelp.php).^28^ Grid parameters of the search space were defined accordingly: grid center coordinates X,Y,Z[43.13, 44.03, 41.23] and grid points X,Y,Z[15, 15, 15] with 1 Å spacing. Preselected compounds from ML models were docked with AutoDock Vina 1.1.2^29^ (exhaustiveness of the global search was set to 10) into the identified binding region.

#### Prioritization of the Identified Hits

Three different classes of compounds were defined after the ML-based screening step: category “G1” (hits from OATP1B1ML-models), category “G2” (hits from OATP1B3ML-models), and category “G3” (hits from OATP2B1ML models). To be able to come up with a shorter list of compounds to be experimentally tested, compounds were first sorted according to their docking score. The top 30 ranked compounds per class (G1, G2, and G3) were kept. Next, physicochemical properties (SlogP, TPSA, SMR, number of rotatable bonds, and AMW) were calculated since these features were previously recognized as important molecular determinants for hepatic OATP activity.^13^ Therefore, our intention was to check whether the newly identified hits fall within the range of known OATP ligands (see Supporting Information Table S2). Ligands with properties falling into the outlier regions were filtered out. In a subsequent filtering step, the chemical diversity of the retained hits was examined as follows: similarities between pairs of compounds were calculated; the size of the maximum common substructure (MCS) of the compound pairs was defined as a similarity metric; a distance matrix was used to hierarchically cluster the compounds; and the complete linkage method was applied to perform hierarchical clustering. Compounds were assigned to a common cluster with the distance threshold of 0.5. A single compound per each cluster was retained. Selection of a single representative per cluster was guided by the docking scores of the respective compounds by taking into account the compounds’ ability to preferentially interact with just one of the three transporters. Finally, 15 compounds per category (G1, G2, and G3) were retained for the final compound set (45 compounds in total; Supporting Information Table S3). Interestingly, none of the 45 selected compounds were also predicted by the general models which motivated us to not include the predictions of this fourth class of compounds.

### Identification of Six Compounds for IC_50_ Value Determination at Multiple Compound Concentrations

The set of 45 compounds was further narrowed down by a manual selection procedure. We based our selection on three criteria: 1) high potency in the single concentration measurements, 2) tendency to show selectivity for one of the transporters, and 3) chemical diversity within the set of the six final hits.

### *In Vitro* Determination of Inhibitory Potential

#### Generation and Maintenance of Cell Lines

A431 cells overexpressing OATP1B1, OATP1B3, or OATP2B1 or their mock transfected controls were generated previously^30^ and maintained in Dulbecco’s modified Eagle medium (DMEM, Gibco, Thermofisher Scientific, Waltham, MA, US) supplemented with 10% fetal bovine serum, 2 mM l-glutamine, 100 units/mL penicillin, and 100 μg/mL streptomycin. Expression and function of OATPs in the cell lines were checked regularly.

#### Transporter Inhibition Measurements

**Figure 2:**
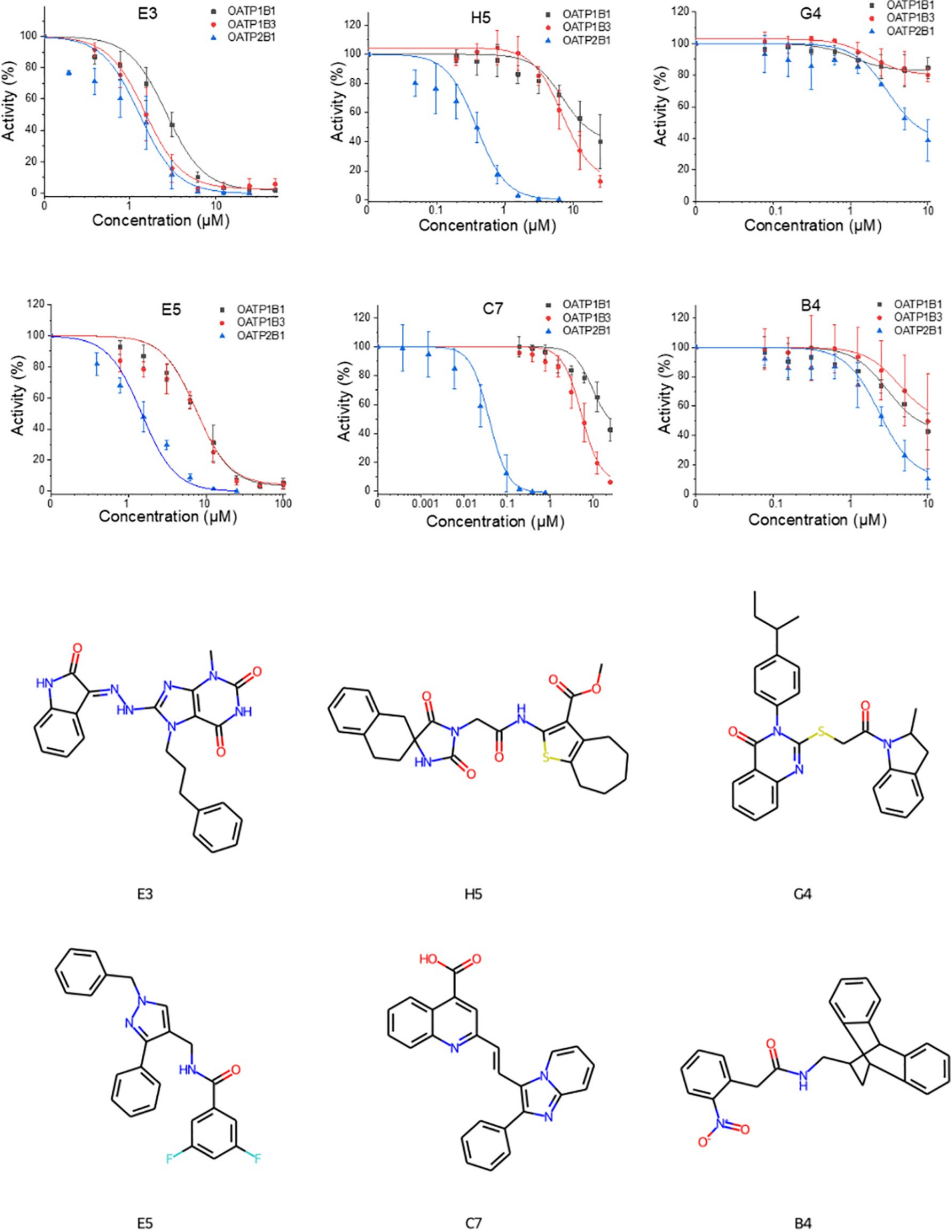
Graphs showing full dose–response curves for the six selected inhibitors (top) and the chemical structures of the respective selected hits (bottom).

Interaction with OATP1B1, OATP1B3, or OATP2B1 was tested in an indirect transport assay using pyranine (8-hydroxypyrene-1,3,6-trisulfonic acid trisodium salt, H1529, Sigma, Merck, Budapest, Hungary) as the test substrate^31,32^ A431 cells overexpressing OATP1B1, OATP1B3, or OATP2B1 or their mock transfected controls were seeded on 96-well plates in a density of 8 × 10^4^ cells per well in 200 μL of cell culture medium 1 day prior to the transport measurements. After 16–24 h, the medium was removed, and the cells were washed three times with 200 μL of PBS (phosphate buffered saline, pH 7.4) and preincubated for 5 min at 37 °C with 50 μL of uptake buffer (125 mM NaCl, 4.8 mM KCl, 1.2 mM CaCl_2_, 1.2 mM KH_2_PO_4_, 12 mM MgSO_4_, 25 mM MES [2-(*N*-morpholino)ethanesulfonic acid, and 5.6 mM glucose, pH 5.5) with or without the tested compound. During the initial screen, the compounds were tested in three different concentrations, 1, 10, or 100 μM, though in some cases due to poor solubility the maximum concentrations were 20 or 50 μM. Each test compound was dissolved in DMSO (that did not exceed 0.5% in samples); solvent controls were also applied. Final hit compounds (*n* = 6) were then tested at eight different concentrations (see Figure 2). The transport reaction was started by the addition of 50 μL of uptake buffer containing pyranine in a final concentration of 10 μM (OATP1B1) or 20 μM (OATP1B3 and OATP2B1), and the cells were further incubated at 37 °C for 15 min (OATP1B1 and OATP2B1) or 30 min (OATP1B3). The reaction was stopped by removing the supernatant. After repeated washing with ice-cold PBS, fluorescence was determined in an Enspire plate reader (PerkinElmer, Waltham, MA) with excitation/emission wavelengths of 460/510 nm. OATP-dependent transport was calculated by extracting fluorescence measured in mock transfected cells and normalized to the fluorescence signal obtained in the absence of the tested compounds (100%). Experiments were repeated in at least three biological replicates.

#### Determining IC_50_ Values

In the first three-point screen, compounds were categorized based on their concentrations needed for 50% inhibition. In the more detailed eight-point inhibition measurements, IC_50_ values were calculated by Hill1 fit, using the Origin Pro 2018 software (OriginLab Corporation, Northampton, MA, USA).

## Results and Discussion

The designed computational strategy–combining ML models with molecular docking–allowed compound selection at various steps.

Two main PCM models were generated with the collected data, one for conformal predictions and one for XGBoost, with the internal CV scores reported in Table 2. These two main models were then modified to either predict compound affinity for each specific protein or for all proteins at the same time. However, this did not change the internal CVs of the PCM models, as the base model was unaltered. In the case of the single transporter models, all internal CVs were reported. First, a general model was created, that would check if a compound was active on all three OATPs at the same time. Then, three selectivity models were created, where the chosen OATP was deemed as active and the remaining two were specifically inactive.

**Table 2:** Internal Cross-Validation Results of the Final CP Models and XGBoost Models^a^

model	ROC value	sensitivity	specificity
CP PCM model	0.762	0.773	0.750
CP OATP1B1	0.773	0.777	**0.769**
CP OATP1B3	0.800	0.833	0.767
CP OATP2B1	0.664	0.726	0.602
XGBoost PCM model	**0.903**	**0.916**	0.702
XGBoost models OATP1B1	0.847	0.867	0.651
XGBoost models OATP1B3	0.872	0.909	0.565
XGBoost models OATP2B1	0.764	0.799	0.627

a Shown are the 5-fold ROC values and the calculated sensitivity and specificity from the confusion matrix. In each column, the highest value is highlighted in bold.

As seen from the internal CV of the various generated ML models (Table 2), they are, in general, performing well for all different modeling tasks and flavors of model building with ROC values between 0.66 (CP model for OATP2B1) and 0.9 (general XGBoost PCM model). Looking a bit more closely at the performances, it becomes obvious that the general models as well as the models for OATP1B1 and OATP1B3 perform better than the ones for OATP2B1 which can be easily explained by the significantly smaller data sets used for training the latter models (an approximately 5 times smaller data set than for the other two transporters; see Table 1). Another thing to note is that the sensitivity for XGBoost models is higher than the CP model and *vice versa* for the specificity. We suspect this is due to the way CP is structured for significance levels, giving more equal metrics across the board; whereas XGBoost likely favors positive predictions as that is the focus of the initial data set. Also, both algorithms (CP and XGBoost) were able to handle the imbalanced nature of the input data sets very well (between approximately 1:2 and 1:5; see Table 2)

### ML-Based Compound Selection

For both QSAR and PCM models, bioactivities were predicted for the constructed Enamine compound set and subsequently ranked on highest confidence of an active compound (QSAR) or highest predicted activity value (PCM). Table 3 summarizes the numbers of predicted active compounds for the filtered Enamine data set by utilizing the 16 different models. Of these, the top 250 were selected from each of the 16 models, wherever possible. These compounds were then pooled together, and any duplicates were removed, which left 3,291 compounds ready for the next selection step.

**Table 3:** Numbers (and Percentages of the Complete Enamine Set of 1,963,425 Compounds) of Predicted Active Compounds Delivered by Each of the 16 Models

method	XGBoost - active (% of total)	CP - active (% of total)
PCM all	64,251 (3.72%)	124,599 (6.35%)
PCM (OATP1B1 only)	76,563 (3.90%)	141 (0.01%)
PCM (OATP1B3 only)	23,909 (1.22%)	0 (0.00%)
PCM (OATP2B1 only)	343 (0.02%)	704,646 (35.89%)
QSAR all	63,140 (3.23%)	193,303 (9.85%)
QSAR (OATP1B1 only)	170,892 (8.73%)	925 (0.05%)
QSAR (OATP1B3 only)	52,011 (2.66%)	1,209 (0.06%)
QSAR (OATP2B1 only)	19,412 (0.99%)	2,047 (0.10%)

There are discrepancies between the two main prediction methods; however, it is difficult to assess which of these methods is better in predicting active compounds, as the amount is not indicative of the quality of these predictions. CP has a lower number of predicted actives in total, with both PCM OATP1B1 and OATP1B3 selective models falling below the threshold of 250 predicted actives (Table 3). Difficulties in identifying actives by the CP-PCM selective models are likely caused by close sequence similarity of OATP1B1 and OATP1B3. As a result, considerably more active compounds were found for OATP2B1. This is the opposite result, as observed for the XGBoost-PCM models, where the two closest proteins are much higher in the number of predicted active compounds compared to OATP2B1 (Table 3). We speculate that in a proteochemometric setting, both gradient boosting and conformal predictions are needed to define compounds of interest.

The single QSAR models seem to work well in a gradient boosting setting but not so well in a conformal prediction setting. The percentages found in the gradient boosting setting are indicative of the amount of information available in the initial training set. The number of predicted actives by the QSAR-CP models seems low, especially compared to the general QSAR model, and they do not follow this training data trend. We theorize that, to date, there is not enough information contained in the OATPs data sets to generate confident selective models.

### Molecular Docking-Based Compound Prioritization

To narrow the field for potential inhibitors further, a docking selection was performed on these 3,291 compounds, as described in the Methods section. After the structure-based VS step, 45 compounds were prioritized (Supporting Information Table S2) on the basis of docking scores and class membership (15 compounds per each class–G1, G2, and G3). Docking scores for the docked compounds were ranging from −10.7 to −2.8 kcal/mol for OATP1B11, from −9.9 to −0.3 kcal/mol for OATP1B3, and from −8.7 to −0.5 kcal/mol for OATP2B1, respectively. For the prioritized list of 45 compounds, docking scores ranged from −9.7 to −8.8 kcal/mol for OATP1B1 (G1 class), from −9.9 to −8.5 kcal/mol for OATP1B3 (G2 class), and from −8.7 to −7.8 kcal/mol for OATP2B1 (G3 class), respectively.

### Experimental Validation

Initial experimental screens (of 44 compounds; one compound had to be excluded because it could not be delivered) detected 36% OATP1B1 (16 actives, 28 inactives), 32% OATP1B3 (14 actives, 27 inactives, 3 activated the transport), and 66% OATP2B1 (29 actives, 15 inactives) compounds with an IC_50_ value ≤10 μM (Table 4). Interestingly, the trends that we observed for the ML-based predictions hold true–our computational strategy seems to be better suited to predict highly active OATP2B1 ligands; although for the training of these models, the smallest data set was available. There are some factors which might have influenced this trend: the OATP2B1 data set was the least imbalanced data set of the three (with an imbalance ratio of ∼1:2), thus its capability to correctly predict the active (minority class) when using the model for external prediction is likely higher than for the models of the other two targets (with a significantly higher imbalance ratio). Another factor might be the smaller chemical diversity of the OATP2B1 training set compounds which likely turned the model into a specialized predictor for a particular set of chemically similar compounds. Computing the average Tanimoto similarity of each of the training set compounds (for every transporter) to the 45 prioritized compounds (on the basis of FCFP_6), indeed a higher similarity was retrieved for OATP2B1 (Tc = 0.3) vs the other two transporters (both Tc = 0.28).

**Table 4:**
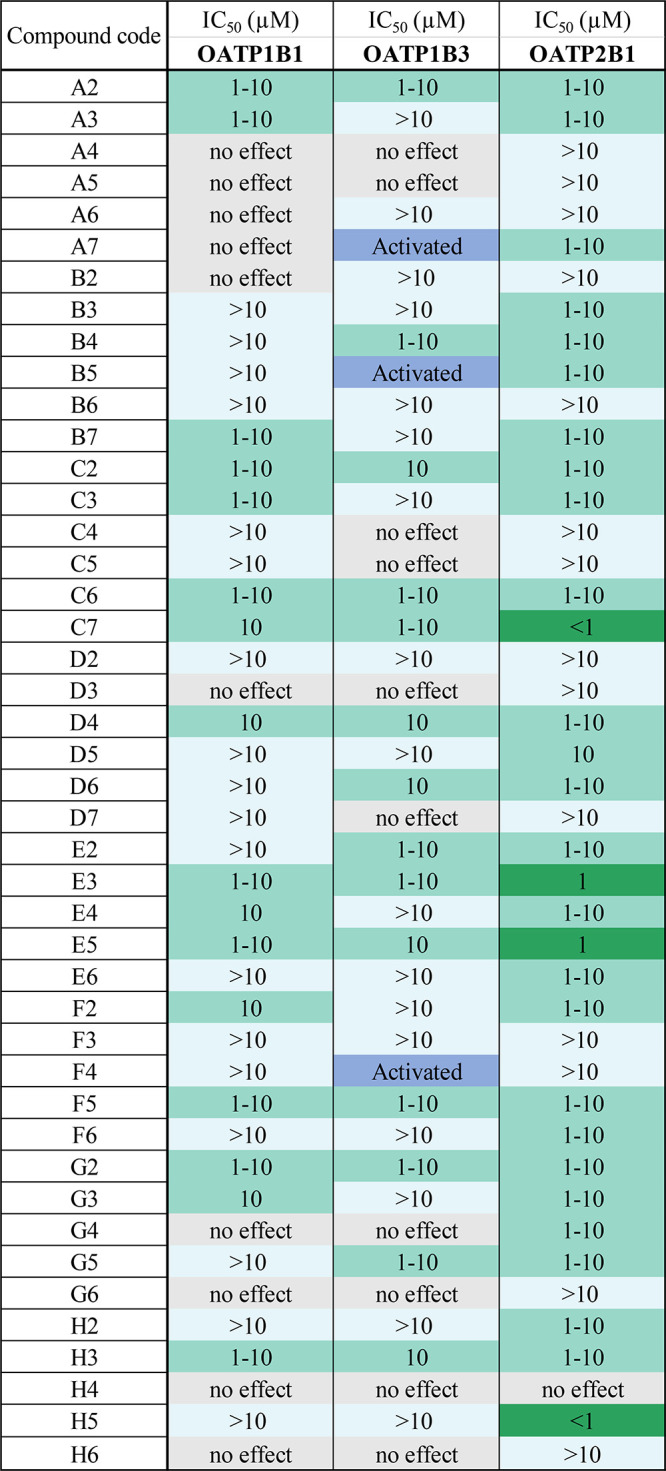
Table Showing the Inhibitory Effect of 44 Compounds Measured in the Initial Screens^a^

a Five categories were determined: 50% inhibition observed below 1 μM, 50% inhibition between 1 and 10 μM, 50% inhibition above 10 μM, and no effect on transport (colored dark gray). In addition, several compounds were identified as transport activators (colored blue).

Finally, OATP2B1 is structurally least similar to the other two transporters which might lead to a competitive advantage over the phylogenetically more similar transporters (OATP1B1 and OATP1B3) when protein information is included in the feature matrix (PCM setting).

The novel OATP data set of 44 compounds includes structures covering a different area of chemical space compared to the structures with OATP bioactivity data gathered from the public domain.^13^ Specifically, newly measured OATP inhibitors are structurally dissimilar at the scaffold level, as evidenced by the comparison of their Murcko scaffolds, compared to previously known ligands (chemical structures of the compounds are depicted in Supporting Information Figure S2).

IC_50_ measurements of the six compounds that were further prioritized by our manual selection procedure (see Methods for details) are listed along with their chemical structures in Figure 2 and Table 5. Strikingly, in this selection, we could identify six (strong) OATP2B1 inhibitors (IC_50_ values ranging from 0.04 to 6 μM), as well as three OATP1B1 inhibitors (2.69 to 10 μM), and five OATP1B3 inhibitors (1.53 to 10 μM) inhibitors. Among these, two novel OATP2B1 inhibitors were discovered (**C7** and **H5**) which show activity comparable to the highest affinity inhibitor reported in the literature–*estra-1,3,5(10)-trien-17-on-2-ylphosphonate* (IC_50_ = 41 nM).^33^ Both compounds do also show a reasonable inhibitory effect on OATP1B3, however, with 18-fold (**H5**) and 135-fold (**C7**) lower affinity. Therefore, particularly, compound **C7** deserves special attention when it comes to the more detailed analysis of molecular interactions. Other compounds measured in transport inhibition experiments showed remarkable selectivity for OATP2B1, whereas with lower affinity toward OATP2B1 (**G4** and **B4**). The remaining two compounds (**E3** and **E5**) did prove to act as pan inhibitors on all three hepatic OATPs.

**Table 5:** IC_50_ Values Determined from Full Dose–Response Curve Measurements for the Six Selected Compounds^a^

IC50 (μM)				
compound identifier	OATP1B1	OATP1B3	OATP2B1	SMILES code
**H5**	∼25	6.95	0.39	COC(=O)c1c2CCCCCc2sc1NC(=O)CN3C(=O)NC4(CCc5ccccc5C4)C3=O
**G4**	no effect	no effect	∼6	CCC(C)c1ccc(cc1)N2C(=Nc3ccccc3C2=O)SCC(=O)N4C(C)Cc5ccccc45
**E5**	7.61	7.50	1.48	Fc1cc(F)cc(c1)C(=O)NCc2cn(Cc3ccccc3)nc2c4ccccc4
**C7**	>10	5.4	**0.04**	OC(=O)c1cc(\C=C\c2c(nc3ccccn23)c4ccccc4)nc5ccccc15
**E3**	**2.69**	**1.53**	1.32	CN1C(=O)NC(=O)c2c1nc(N\N=C/3\C(=O)Nc4ccccc34)n2CCCc5ccccc5
**B4**	∼10	∼10	2.37	[O][N+](=O)c1ccccc1CC(=O)NCC2CC3c4ccccc4C2c5ccccc35

a Best values for each transporter are shown in bold.

### Insights from Molecular Docking

For each transporter, three to four possible binding sites were identified via FTMap server, as described in the Methods section. Predicted binding sites are visually depicted in Supporting Information Figure S3. Interestingly, binding cavities in all three transporters were found in the same region, lined by TMH1, TMH2, TMH4, TMH5, TMH7, TMH8, and TMH11. Concrete residues belonging to this region in the three transporters are listed in Table 6.

**Table 6:** Amino Acid Residues Contributing to the Predicted Binding Site in Each Respective Transmembrane Helix (TM)

TM	OATP1B1	OATP1B3	OATP2B1
2	LEU78	LEU78	THR99
4	VAL189	VAL189	GLN207
5	ASN213	ASN213	PHE231
5	ALA216	GLY216	THR234
5	MET217	MET217	MET235
7	GLN348	GLN348	LEU383
7	VAL349	VAL349	SER384
7	TYR352	PHE352	ALA387
7	PHE356	PHE356	ALA391
8	ILE385	THR385	SER420
10	ALA549	ALA549	CYS576
10	GLY552	GLY552	HIS579
11	MET577	MET577	MET604
11	ARG580	ARG580	ARG607

Docking poses and the respective protein environments of the active subset of the prioritized 44 compounds (16 OATP1B1 actives, 14 OATP1B3 actives, and 29 OATP2B1 actives when considering activity cutoff ≤10 μM) were examined in more detail in order to gain insights into driving factors for activity (and potentially also selectivity) at a molecular level.

**Figure 3:**
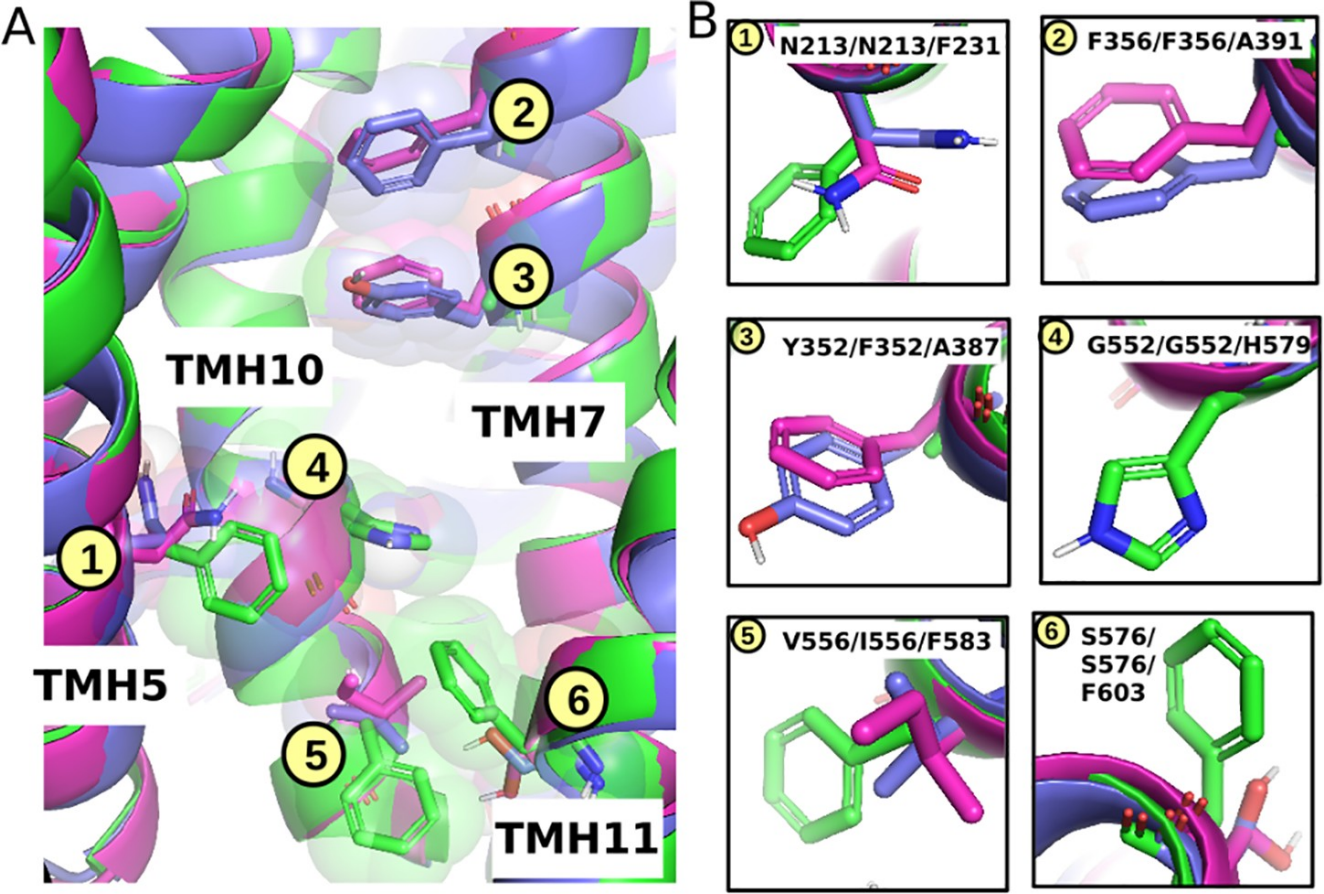
Localization of aromatic residues in OATP1B1 (blue structure), OATP1B3 (magenta structure), and OATP2B1 (green structure) impacts pocket geometry and accessibility. (A) Side view of TMH5, 7, 10, and 11. (B) Specific amino acid residues in a close-up view.

Volumetric maps show a remarkable difference in the localization of aromatic residues when comparing the binding sites of OATP1B1/OATP1B3 and OATP2B1 (Supporting Information Figure S4). By closer inspection of the respective binding regions (lined by TMH5, TMH7, TMH10, and TMH11), several replacements of aromatic to aliphatic residues can be observed in OATP1B1/OATP1B3 compared to OATP2B1 and *vice versa* (see Figure 3). Specifically, TYR352/PHE352 in OATP1B1/OATP1B3 at TMH7 are replaced by ALA387 in OATP2B1, PHE356 in OATP1B1/OATP1B3 at TMH7 is replaced by ALA391 in OATP2B1, and GLY552 in OATP1B1/OATP1B3 at TMH10 is replaced by HIS579 in OATP2B1. Other amino acid substitutions include ASN213 in OATP1B1/OATP1B3 at TMH5 being replaced by PHE231 in OATP2B1, VAL556/ILE556 in OATP1B1/OATP1B3 at TMH10 being replaced by PHE583 in OATP2B1, and SER576 in OATP1B1/OATP1B3 at TMH11 being replaced by PHE603.

**Figure 4:**
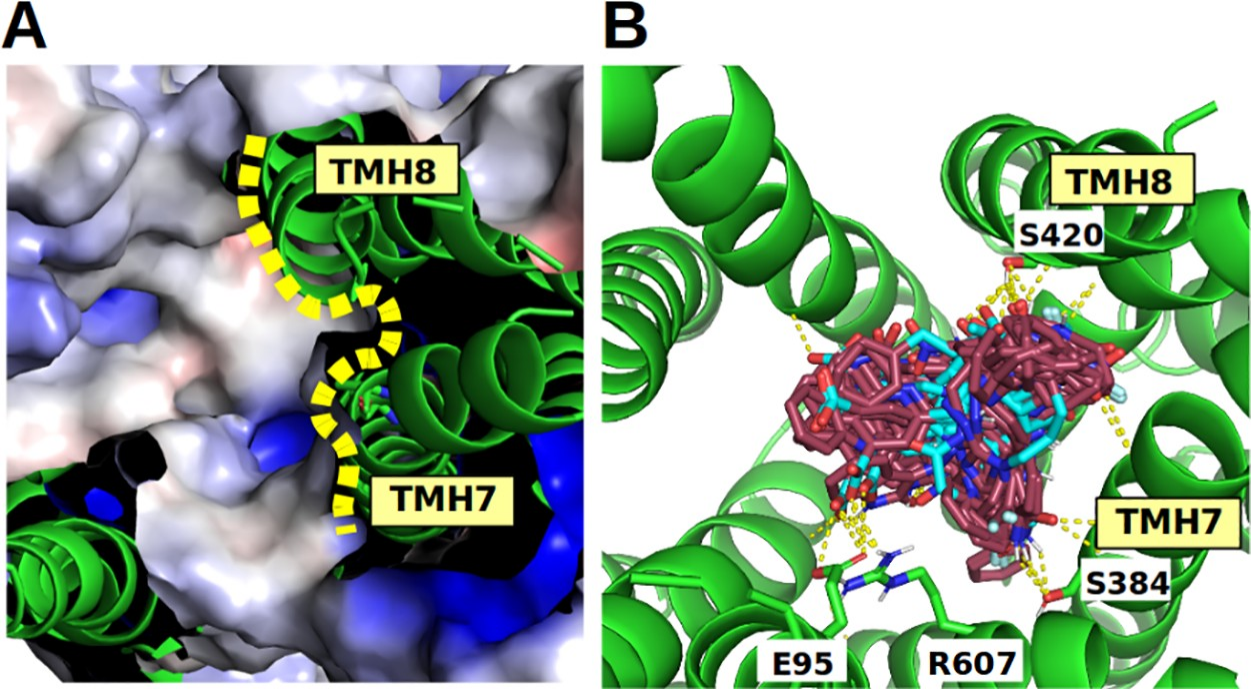
(A) Mapped electrostatic potential shows an accessible cavity between TMH7 and TMH8 in OATP2B1. (B) Docked poses (top view) for the most potent OATP2B1 actives (codes: **B4**, **C7**, **E3**, and **E5**) show shape complementarity to the OATP2B1 binding site. Several residues (SER420 or SER384) in TMH7 and TMH8 form H-bonds with the docked ligands (indicated by yellow dashed line). In addition, H-bonds are formed with E95 (TMH2) and R607 (TMH11). HIS579 interacts with the presented ligands in the lower part of the central binding region and hence is not visible in this visualization.

Calculation of the electrostatic potential and mapping the surface onto the binding site show the substitutions at TMH7 that are crucial for ligands to become partially accommodated in the subcavity located in the C-terminal domain (Figure 4). Accession of the C-terminal subcavity in OATP1B1 and OATP1B3 is blocked due to the presence of aromatic residues at positions 352 (TYR/PHE) and 356 (PHE). In contrast, the electrostatic surface of OATP2B1 shows a small region at the TMH7/TMH8 interface which can be accessed from the central cavity of the transporter (Figure 4 and Supporting Information Figure S5). Indeed, the strong OATP2B1 inhibitors identified in this study do structurally fit into the accessible surface in OATP2B1 (Figure 4). Further, the replacement of HIS579 in OATP2B1 at TMH10 to GLY552 in OATP1B1/OATP1B3 has an additional effect on ligand recognition in the C-terminal domain, as it further restricts the space where ligands can bind to. Since HIS579 in OATP2B1 is pointing toward the center of the transporter cavity and thus restricts the translocation pore of the transporter, it raises the question whether HIS579 could adopt different rotameric states, which, in turn, could have a significant impact on ligand binding. Therefore, rotamer analysis was performed to model alternative side chain orientations of HIS579. The probability of adopting different rotamers seems low due to observed steric clashes with neighboring residues (see Supporting Information Figure S6). We therefore conclude that our structural model for OATP2B1 is very likely depicting the correct orientation of HIS579.

Calculation of protein–ligand interaction fingerprints (PLIFs) led to the identification of key residues which interact with the newly measured compounds (Supporting Information Figures S7–S9). The most frequent residues in OATP1B1 (ASN213, MET217, GLN348, ALA549, GLY552, ARG580) and OATP1B3 (VAL189, ASN213, MET217, GLN348, ALA549, GLY552, MET577, ARG580) are largely overlapping. The very high similarity of protein–ligand interactions between the two transporters might be attributed to their high sequence similarity (∼80%). Interestingly, some of these residues also appeared to be implicated in the binding of steroid analogs as reported in our previous paper.^12^ These findings provide a consistent picture about the structural determinants of ligand recognition in the central cavity, as already shown in our previous study for OATP1B1 and OATP1B3 transporters.^12^ However, OATP2B1 shows molecular interactions with different residues, such as MET235, SER384, SER420, SER572, ALA575, CYS576, HIS579, and ARG607. Interestingly, the majority of the frequently interacting residues in OATP2B1 is nonconserved across the three hepatic OATP members (such as SER420, CYS576, and HIS579). HIS579 has already been confirmed by mutational experiments to be crucial for the OATP2B1 substrate transport.^34^

The novel, highly active hepatic OATP ligands (see Table 5) were studied in further detail with respect to their binding modes in the respective transporter(s). Since all six compounds show inhibitory activity on OATP2B1 (ranging from 0.04 μM for compound **C7** to ∼6 μM for compound **G4**), we first analyzed docking poses of all ligands in OATP2B1. In general, all ligands are accommodated in a way that one end of the ligand is stabilized at the TMH7/TMH8 interface, while the other end of the ligand is tilted via a flexible linker to reach the interface between TMH7 and TMH11 in an “L-shaped” fashion (Figure 4B and Supporting Information Figure S10). Furthermore, compounds **B4** and **E3** form π–π interactions (parallel-displaced type) with HIS579 (Supporting Information Figure S10). For other compounds investigated here, HIS579 does not directly form π–π interactions but rather acts as a mechanical barrier that disables the ligand to get bound more deeply to the inner (cytoplasmic) part of the C-terminal binding site. Interestingly, mutations of HIS579 led to an altered uptake of estrone-3-sulfate, pravastatin, rosuvastatin, and sulfasalazine, as demonstrated by Hoshino et al. in 2016.^34^

Further, the six compounds can be categorized into three different activity classes as follows: category ***(1)*** is pan inhibitors (compounds **E3** and **E5**); category ***(2)*** is dual OATP1B3/OATP2B1 inhibitors (compounds **H5** and **C7**) which at the same time happen to be the strongest OATP2B1 inhibitors identified in this study; and category ***(3)*** is OATP2B1 selective inhibitors (compounds **B4** and **G4**). It is noteworthy that we were unable to find compounds showing preferential inhibition for OATP1B1 or OATP1B3.

**Figure 5:**
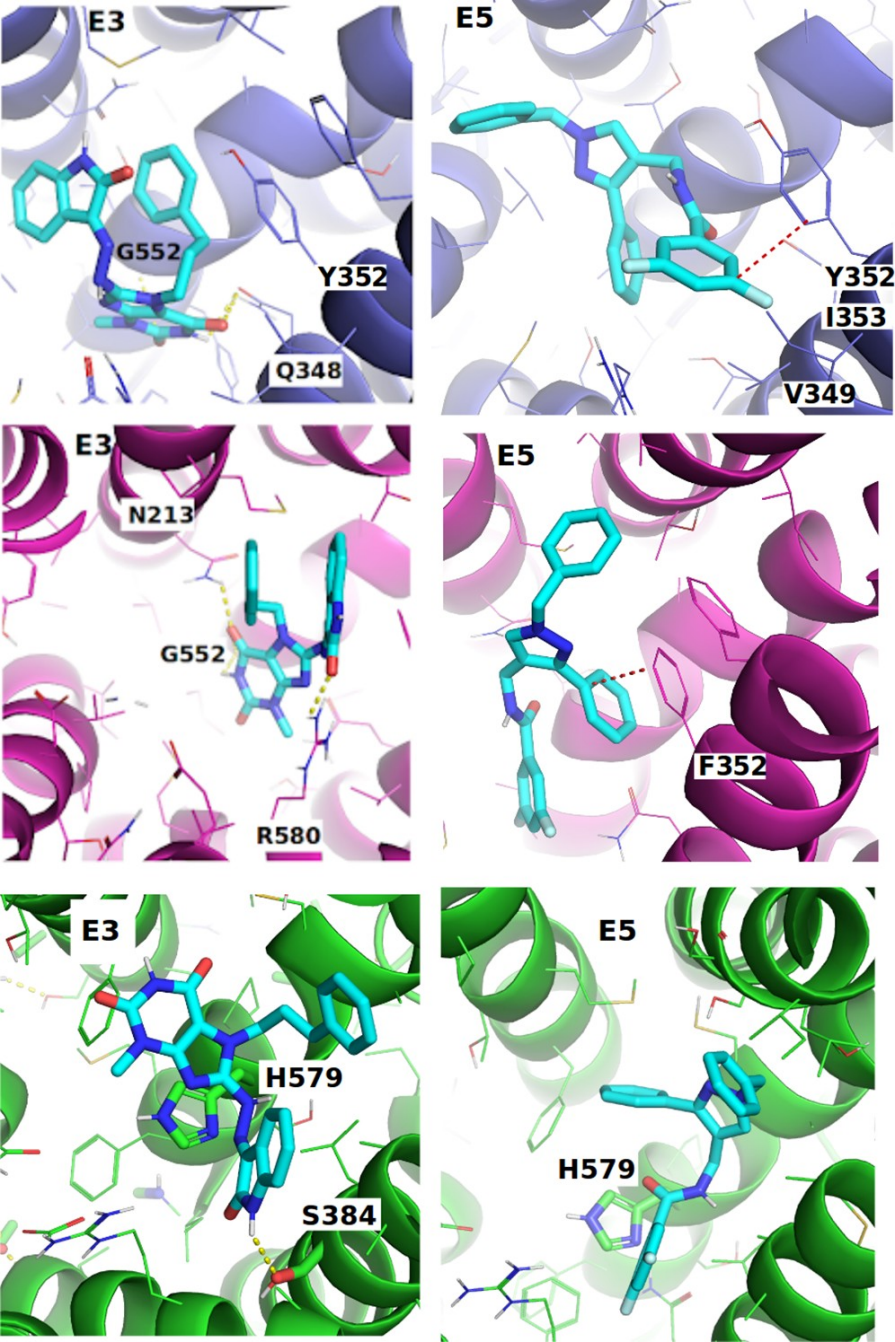
Prominent interactions of compounds **E3** and **E5** in OATP1B1 (blue structure), OATP1B3 (magenta structure), and OATP2B1 (green structure). Hydrogen bonds are indicated by yellow dashed lines. Poses shown here represent the most populated poses per compound identified by hierarchical pose clustering.

Category ***(1)*** of active compounds with pan inhibitory activity (compounds **E3** and **E5**) shows a more or less consistent binding behavior in OATP1B1 and OATP1B3 being positioned below TYR352/PHE352. Compound **E3** interacts with GLY552 and GLN348 (OATP1B1) and ARG580 (OATP1B3) and shows formation of intramolecular π–π interactions which probably increases ligand stability in the binding pocket. For OATP2B1, compound **E3** shows a π–π interaction with HIS579 and a hydrogen bond interaction with SER384 (Figure 5 and Supporting Information Figure S11). Interestingly, GLY552, GLN348, and ARG580 have already been shown to be implicated in steroid analog binding in our previous study,^12^ and ARG580 has been shown previously to be implicated in transport activity of both OATP1B1^35^ and OATP1B3.^36^ The intramolecular interaction in compound **E3** was also found in OATP2B1, albeit not in the most populated pose cluster.

Compound **E5** shows greater differences in bioactivity values (7.61 μM for OATP1B1, 7.50 μM for OATP1B3, and 1.48 μM for OATP2B1) which might be explained by a mechanical stabilization effect through interaction with TYR352 in OATP1B1 and PHE352 in OATP1B3, as well as by HIS579 in OATP2B1. In OATP2B1, compound **E5** is shifted more toward the upper part of the C-terminal domain due to the different constitution of aromatic and small residues in TMH7 (Figure 3 and Supporting Information Figure S11B). In OATP1B1 and OATP1B3, however, compound **E5** is bound in such a way that its plane aromatic ring is mechanically stabilized by TYR352 (face to face interaction), while the fluoro substituents reach into the hydrophobic region lined by ILE353 and VAL349 (Figure 5). Interestingly, ILE353THR substitution is a known single nucleotide polymorphism that leads to a decrease in OATP1B1 activity.^37^

Category ***(2)*** compounds **H5** and **C7** are dual inhibitors of OATP1B3 and OATP2B1. In addition, compound **C7** is also the strongest OATP2B1 inhibitor of this study (40 nM), and therefore, it is of particular interest to study binding poses. Hydrogen bond formation between SER420 in OATP2B1 and compound **C7** and an additional H-bond with GLN207 (replaced by VAL189 in OATP1B1 and OATP1B3) was observed. Interestingly, SER420 in OATP2B1 is replaced by THR385 in OATP1B3 and by ILE385 in OATP1B1. Although, in our study, no direct interaction of compound **C7** with OATP1B3 was observed, we hypothesize that THR385 could adopt a corresponding hydrogen bond interaction with the ligand, given its chemical similarity with SER420; whereas the presence of ILE385 in OATP1B1 increases the hydrophobicity of the binding pocket and disables formation of hydrogen bonds. Similarly, compound **H5** shows hydrogen bond interactions with GLN207 and SER420 in OATP2B1, thus leading to the same hypothesis, as in the case of compound **C7** (Supporting Information Figure S12).

Compounds **B4** and **G4** belong to category ***(3)*** of “selective” OATP2B1 ligands where compound **G4** shows no effect on OATP1B1 and OATP1B3 with moderate activity on OATP2B1 (around 6 μM), while compound **B4** shows stronger activity on OATP2B1 (2.4 μM) but borderline activity on OATP1B1 and OATP1B3 as well (around 10 μM). For both compounds, a consistent binding pattern in OTP2B1 is observed showing hydrogen bond interactions with SER420 (ILE385 in OATP1B1 and THR385 in OATP1B3) and additionally an interaction with HIS579 (GLY552 in OATP1B1/OATP1B3). It seems likely that ligand selectivity can be attributed to the additional interaction with HIS579 which is lacking in the case of the other two transporters (where HIS579 is replaced by GLY552). In addition, HIS579 acts as a mechanical barrier forcing the ligand to be accommodated in the upper half of OATP2B1.

## Summary and Conclusions

*In silico* identification of novel OATP inhibitors confirmed by experimental validation is a promising approach which can be exploited to guide the design of novel chemical probes. Such compounds can be used as tools to study the physiological role of these clinically important transporters. In this study, the diverse REAL drug-like set was initially screened by a combination of different machine learning models including proteochemometric models and conformal prediction models. By consensus ranking of the identified hits from the ligand-based screening, 3,291 compounds could be identified that were further docked into the OATP1B1, OATP1B3, and OATP2B1 structural models to prioritize 44 compounds for subsequent transporter inhibition assay experiments. By this procedure, 29 new active compounds (activity threshold ≤10 μM) with either selective, dual, or pan inhibitory activity were identified. Interestingly, the strongest OATP2B1 inhibitor (compound **C7**, IC_50_ = 40 nM) shows similar affinity to the strongest OATP2B1 inhibitor that was recently reported in the literature.^33^ These findings indicate that the developed integrative modeling pipeline, combining AI-based and structure-based methods, is capable of identifying highly active compounds. Interestingly, our computational pipeline seems better suited to predict OATP2B1 inhibitors since it detected twice as many hits for OATP2B1 vs the other two transporters. One of the reasons might be the higher phylogenetic difference of OATP2B1 to the other two transporters which is included in the proteochemometric descriptors used for some of the ML models. While we can observe that the PCM produces less hits in the virtual screen and has a lower ROC value in CV, it would seem that the resulting model has higher predictive capabilities.

Docking poses of the novel OATP inhibitors were closely inspected in order to delineate potential molecular reasons for ligand interaction and selectivity. A remarkable difference comparing the constitution of the OATP1B1/OATP1B3 vs OATP2B1 binding sites was detected. It is characterized by diverging localization of aromatic residues in the inner cavity extending into the C-terminal domain. We have shown that the identified “L-shaped” inhibitors fit well into the OATP2B1 binding site as a result of their shape complementarity. Overall, the study presented here delivers novel OATP inhibitors with various OATP overlapping profiles, providing molecular insight into the C-terminal binding region of hepatic OATPs.

## Data and Software Availability

All data and code needed to reproduce the conclusions of this study are available from an open GitHub repository: https://github.com/AlzbetaTuerkova/VirtualScreening.

## Supporting Information Available

Pairwise sequence identity/similarity of hepatic OATPs calculated via Emboss NEEDLE algorithm; range of physicochemical properties for known (published) OATP1B1, OATP1B3, and OATP2B1 ligands; compounds belonging to category “G1” (potentially OATP1B1 selective compounds), category “G2” (potentially OATP1B3 selective compounds), and category “G3” class (potentially OATP2B1 selective compounds), respectively; Venn diagram showing overlap of active compounds for OATP1B1, OATP1B3, and OATP2B1; chemical structures of compound set (*n* = 44) prioritized by ML-based virtual screening; predicted binding sites for OATP1B1, OATP1B3, and OATP2B1, respectively; volumetric map showing distribution of aromatic residues in binding site of OATP1B1, OATP1B3, and OATP2B1 and superimposed transporters; electrostatic potential mapped in OATP1B1, OATP1B3, and OATP2B1 inner cavities; possible rotamers for HIS579 in OATP2B1; protein–ligand interaction fingerprints for newly identified OATP1B1 inhibitors; protein–ligand interaction fingerprints for newly identified OATP1B3 inhibitors; protein–ligand interaction fingerprints for newly identified OATP2B1 inhibitors; docking poses of OATP2B1 inhibitors (compounds **B4**, **C7**, **E****3**, **E5**, **H5**, and **G4**); 3D structures of compounds **E3** and **E5**, respectively, and docking poses of compounds **E3** and **E**5 in OATP1B1, OATP1B3, and OATP2B1 transporters, respectively; and docking poses for compounds **H5** and **C7** in OATP2B1 showing hydrogen bond interaction with SER420 (PDF)

## Notes

The authors of this manuscript are committed to supporting and advancing women in chemistry. More than half of the authors on this manuscript are women (five out of ten) with two out of four of the research group leaders contributing to this work being female.

## Abbreviations

AMW: average molecular weight
CP: conformal prediction
FCFP: functional connection fingerprints
ITC: International Transporter Consortium
KNIME: Konstanz Information Miner
MCS: maximum common substructure
MFS: major facilitator superfamily
ML: machine learning
OATP: organic anion transporting polypeptide
PCM: proteochemometric modeling
PDB: Protein Data Bank
PLIF: protein ligand interaction fingerprint
QSAR: quantitative structure activity relationship
RF: Random Forest
ROC: receiver operator characteristic, SASA, solvent accessible surface area
SMR: molecular refractivity
TMH: transmembrane helix
TPSA: topological polar surface area

## References

[ref1] Karlgren M.; Vildhede A.; Norinder U.; Wisniewski J. R.; Kimoto E.; Lai Y.; Haglund U.; Artursson P. Classification of Inhibitors of Hepatic Organic Anion Transporting Polypeptides (OATPs): Influence of Protein Expression on Drug-Drug Interactions. J. Med. Chem. 2012, 55, 4740–4763. 10.1021/jm300212s. 22541068 PMC3361267

[ref2] Ismair M. G.; Stieger B.; Cattori V.; Hagenbuch B.; Fried M.; Meier P. J.; Kullak-Ublick G. A. Hepatic Uptake of Cholecystokinin Octapeptide by Organic Anion-Transporting Polypeptides OATP4 and OATP8 of Rat and Human Liver. Gastroenterology 2001, 121, 1185–1190. 10.1053/gast.2001.28704. 11677211

[ref3] Kullak-Ublick G. A.; Stieger B.; Meier P. J. Enterohepatic Bile Salt Transporters in Normal Physiology and Liver Disease. Gastroenterology 2004, 126, 322–342. 10.1053/j.gastro.2003.06.005. 14699511

[ref4] Keppler D. The Roles of MRP2, MRP3, OATP1B1, and OATP1B3 in Conjugated Hyperbilirubinemia. Drug Metab. Dispos. 2014, 42, 561–565. 10.1124/dmd.113.055772. 24459177

[ref5] van de Steeg E.; Stránecký V.; Hartmannová H.; Nosková L.; Hřebíček M.; Wagenaar E.; Esch A. van; Waart D. R. de; Elferink R. P. J. O.; Kenworthy K. E.; Sticová E.; al-Edreesi M.; Knisely A. S.; Kmoch S.; Jirsa M.; Schinkel A. H. Complete OATP1B1 and OATP1B3 Deficiency Causes Human Rotor Syndrome by Interrupting Conjugated Bilirubin Reuptake into the Liver. J. Clin. Invest. 2012, 122, 519–528. 10.1172/JCI59526. 22232210 PMC3266790

[ref6] Giacomini K. M.; Huang S. M.; Tweedie D. J.; Benet L. Z.; Brouwer K. L.; Chu X.; Dahlin A.; Evers R.; Fischer V.; Hillgren K. M.; Hoffmaster K. A.; Ishikawa T.; Keppler D.; Kim R. B.; Lee C. A.; Niemi M.; Polli J. W.; Sugiyama Y.; Swaan P. W.; Ware J. A.; Wright S. H.; Yee S. W.; Zamek-Gliszczynski M. J.; Zhang L. Membrane Transporters in Drug Development. Nat. Rev. Drug Discovery 2010, 9, 215–236. 10.1038/nrd3028. 20190787 PMC3326076

[ref7] Maia E. H. B.; Assis L. C.; de Oliveira T. A.; da Silva A. M.; Taranto A. G. Structure-Based Virtual Screening: From Classical to Artificial Intelligence. Front. Chem. 2020, 8, 343 10.3389/fchem.2020.00343. 32411671 PMC7200080

[ref8] Kimber T. B.; Chen Y.; Volkamer A. Deep Learning in Virtual Screening: Recent Applications and Developments. Int. J. Mol. Sci. 2021, 22, 4435 10.3390/ijms22094435. 33922714 PMC8123040

[ref9] De Bruyn T.; Westen G. J. P. van; Ijzerman A. P.; Stieger B.; Witte P. de; Augustijns P. F.; Annaert P. P. Structure-Based Identification of OATP1B1/3 Inhibitors. Mol. Pharmacol. 2013, 83, 1257–1267. 10.1124/mol.112.084152. 23571415

[ref10] Khuri N.; Zur A. A.; Wittwer M. B.; Lin L.; Yee S. W.; Sali A.; Giacomini K. M. Computational Discovery and Experimental Validation of Inhibitors of the Human Intestinal Transporter OATP2B1. J. Chem. Inf. Model. 2017, 57, 1402–1413. 10.1021/acs.jcim.6b00720. 28562037

[ref11] Wishart D. S.; Feunang Y. D.; Guo A. C.; Lo E. J.; Marcu A.; Grant J. R.; Sajed T.; Johnson D.; Li C.; Sayeeda Z.; Assempour N.; Iynkkaran I.; Liu Y.; Maciejewski A.; Gale N.; Wilson A.; Chin L.; Cummings R.; Le D.; Pon A.; Knox C.; Wilson M. DrugBank 5.0: A Major Update to the DrugBank Database for 2018. Nucleic Acids Res. 2018, 46, D1074–D1082. 10.1093/nar/gkx1037. 29126136 PMC5753335

[ref12] Tuerkova A.; Ungvári O.; Laczkó-Rigó R.; Mernyák E.; Szakács G.; Özvegy-Laczka C.; Zdrazil B. Data-Driven Ensemble Docking to Map Molecular Interactions of Steroid Analogs with Hepatic Organic Anion Transporting Polypeptides. J. Chem. Inf. Model. 2021, 61, 3109–3127. 10.1021/acs.jcim.1c00362. 34105971 PMC8243326

[ref13] Türková A.; Jain S.; Zdrazil B. Integrative Data Mining, Scaffold Analysis, and Sequential Binary Classification Models for Exploring Ligand Profiles of Hepatic Organic Anion Transporting Polypeptides. J. Chem. Inf. Model. 2019, 59, 1811–1825. 10.1021/acs.jcim.8b00466. 30372058 PMC6541895

[ref14] Pei J.; Grishin N. V. PROMALS3D: Multiple Protein Sequence Alignment Enhanced with Evolutionary and Three-Dimensional Structural Information. Methods Mol. Biol. Clifton NJ. 2014, 1079, 263–271. 10.1007/978-1-62703-646-7_17. PMC4506754 24170408

[ref15] McGuffin L. J.; Bryson K.; Jones D. T. The PSIPRED Protein Structure Prediction Server. Bioinformatics 2000, 16, 404–405. 10.1093/bioinformatics/16.4.404. 10869041

[ref16] Viklund H.; Elofsson A. OCTOPUS: Improving Topology Prediction by Two-Track ANN-Based Preference Scores and an Extended Topological Grammar. Bioinformatics 2008, 24, 1662–1668. 10.1093/bioinformatics/btn221. 18474507

[ref17] Vovk V.; Gammerman A.; Shafer G. Algorithmic Learning in a Random World; Springer Science & Business Media; 2005; 10.1007/b106715.

[ref18] Norinder U.; Carlsson L.; Boyer S.; Eklund M. Introducing Conformal Prediction in Predictive Modeling. A Transparent and Flexible Alternative to Applicability Domain Determination. J. Chem. Inf. Model. 2014, 54, 1596–1603. 10.1021/ci5001168. 24797111

[ref19] Breiman L. Random Forests. Mach. Learn. 2001, 45, 5–32. 10.1023/A:1010933404324.

[ref20] Pedregosa F.; Varoquaux G.; Gramfort A.; Michel V.; Thirion B.; Grisel O.; Blondel M.; Prettenhofer P.; Weiss R.; Dubourg V.; Vanderplas J.; Passos A.; Cournapeau D.; Brucher M.; Perrot M.; Duchesnay É. Scikit-Learn: Machine Learning in Python. J. Mach. Learn. Res. 2011, 12, 2825–2830.

[ref21] Linusson H. Nonconformist; 2022.

[ref22] Carlsson L.; Eklund M.; Norinder U. Aggregated Conformal Prediction. In Artificial Intelligence Applications and Innovations; Iliadis L., Maglogiannis I., Papadopoulos H., Sioutas S., Makris C., Eds.; IFIP Advances in Information and Communication Technology; Springer: Berlin, Heidelberg, 2014; pp 231–240, 10.1007/978-3-662-44722-2_25.

[ref23] Rogers D.; Hahn M. Extended-Connectivity Fingerprints. J. Chem. Inf. Model. 2010, 50, 742–754. 10.1021/ci100050t. 20426451

[ref24] Sandberg M.; Eriksson L.; Jonsson J.; Sjöström M.; Wold S. New Chemical Descriptors Relevant for the Design of Biologically Active Peptides. A Multivariate Characterization of 87 Amino Acids. J. Med. Chem. 1998, 41, 2481–2491. 10.1021/jm9700575. 9651153

[ref25] Hellberg S.; Sjoestroem M.; Skagerberg B.; Wold S. Peptide Quantitative Structure-Activity Relationships, a Multivariate Approach. J. Med. Chem. 1987, 30, 1126–1135. 10.1021/jm00390a003. 3599020

[ref26] Shivanyuk A. N.; Ryabukhin S. V.; Tolmachev A.; Bogolyubsky A. V.; Mykytenko D. M.; Chupryna A. A.; Heilman W.; Kostyuk A. N. Enamine Real Database: Making Chemical Diversity Real. Chem. Today 2007, 25, 58.

[ref27] Wagner J. R.; Sørensen J.; Hensley N.; Wong C.; Zhu C.; Perison T.; Amaro R. E. POVME 3.0: Software for Mapping Binding Pocket Flexibility. J. Chem. Theory Comput. 2017, 13, 4584–4592. 10.1021/acs.jctc.7b00500. 28800393 PMC5751414

[ref28] Kozakov D.; Grove L. E.; Hall D. R.; Bohnuud T.; Mottarella S. E.; Luo L.; Xia B.; Beglov D.; Vajda S. The FTMap Family of Web Servers for Determining and Characterizing Ligand-Binding Hot Spots of Proteins. Nat. Protoc. 2015, 10, 733–755. 10.1038/nprot.2015.043. 25855957 PMC4762777

[ref29] Trott O.; Olson A. J. AutoDock Vina: Improving the Speed and Accuracy of Docking with a New Scoring Function, Efficient Optimization, and Multithreading. J. Comput. Chem. 2009, 31, 455–461. 10.1002/jcc.21334. PMC3041641 19499576

[ref30] Patik I.; Székely V.; Német O.; Szepesi Á.; Kucsma N.; Várady G.; Szakács G.; Bakos É.; Özvegy-Laczka C. Identification of Novel Cell-Impermeant Fluorescent Substrates for Testing the Function and Drug Interaction of Organic Anion-Transporting Polypeptides, OATP1B1/1B3 and 2B1. Sci. Rep. 2018, 8, 2630 10.1038/s41598-018-20815-1. 29422623 PMC5805760

[ref31] Székely V.; Patik I.; Ungvári O.; Telbisz Á.; Szakács G.; Bakos É.; Özvegy-Laczka C. Fluorescent Probes for the Dual Investigation of MRP2 and OATP1B1 Function and Drug Interactions. Eur. J. Pharm. Sci. 2020, 151, 105395 10.1016/j.ejps.2020.105395. 32473861

[ref32] Mohos V.; Fliszár-Nyúl E.; Ungvári O.; Bakos É.; Kuffa K.; Bencsik T.; Zsidó B. Z.; Hetényi C.; Telbisz Á.; Özvegy-Laczka C.; Poór M. Effects of Chrysin and Its Major Conjugated Metabolites Chrysin-7-Sulfate and Chrysin-7-Glucuronide on Cytochrome P450 Enzymes and on OATP, P-Gp, BCRP, and MRP2 Transporters. Drug Metab. Dispos. 2020, 48, 1064–1073. 10.1124/dmd.120.000085. 32661014

[ref33] Jójárt R.; Laczkó-Rigó R.; Klement M.; Köhl G.; Kecskeméti G.; Özvegy-Laczka C.; Mernyák E. Design, Synthesis and Biological Evaluation of Novel Estrone Phosphonates as High Affinity Organic Anion-Transporting Polypeptide 2B1 (OATP2B1) Inhibitors. Bioorganic Chem. 2021, 112, 104914 10.1016/j.bioorg.2021.104914. 33932771

[ref34] Hoshino Y.; Fujita D.; Nakanishi T.; Tamai I. Molecular Localization and Characterization of Multiple Binding Sites of Organic Anion Transporting Polypeptide 2B1 (OATP2B1) as the Mechanism for Substrate and Modulator Dependent Drug-Drug Interaction. MedChemComm 2016, 7, 1775–1782. 10.1039/C6MD00235H.

[ref35] Miao Y.; Hagenbuch B. Conserved Positively Charged Amino Acid Residues in the Putative Binding Pocket Are Important for OATP1B1 Function. FASEB J. 2007, 21, A196–A197. 10.1096/fasebj.21.5.A196-d.

[ref36] Glaeser H.; Mandery K.; Sticht H.; Fromm M.; König J. Relevance of Conserved Lysine and Arginine Residues in Transmembrane Helices for the Transport Activity of Organic Anion Transporting Polypeptide 1B3. Br. J. Pharmacol. 2010, 159, 698–708. 10.1111/j.1476-5381.2009.00568.x. 20100277 PMC2828033

[ref37] Tirona R. G.; Leake B. F.; Merino G.; Kim R. B. Polymorphisms in OATP-C: identification of multiple allelic variants associated with altered transport activity among European- and African-Americans. J. Biol. Chem. 2001, 276, 35669–35675. 10.1074/jbc.M103792200. 11477075

