# Supplementary material for: Identifying Novel Inhibitors for Hepatic Organic Anion Transporting Polypeptides by Machine Learning-Based Virtual Screening
Source: J Chem Inf Model. 2022 Mar 11;62(24):6323–35. doi: 10.1021/acs.jcim.1c01460 (PMC9795544; doi:10.1021/acs.jcim.1c01460)
Supplement: Supplementary file 1 — ci1c01460_si_001.pdf [file ci1c01460_si_001.pdf]

Supporting Information

Identifying Novel Inhibitors for Hepatic Organic Anion Transporting Polypeptides by Machine Learning-Based Virtual Screening

Alzbeta Tuerkova,[1,$] Brandon J. Bongers,[2,$] Ulf Norinder,[3,4] Orsolya Ungvári,[5,6] Virág Székely,[5] Andrey Tarnovskiy,[7] Gergely Szakács,[5,8] Csilla Özvegy-Laczka,[5] Gerard J.P. van Westen[2,] and Barbara Zdrazil[1,]**

[1] Department of Pharmaceutical Sciences, Division of Pharmaceutical Chemistry, University of Vienna, Althanstraße 14, A-1090 Vienna, Austria

[2] Division of Drug Discovery and Safety, Leiden Academic Centre for Drug Research, Leiden University, P.O. Box 9502, 2300 RA, Leiden, The Netherlands

[3] Department of Pharmaceutical Biosciences, Uppsala University, Box 591, SE-75124, Uppsala Sweden,

[4] MTM Research Centre, School of Science and Technology, Örebro University, SE-70182 Örebro, Sweden

[5] Drug resistance Research Group, Institute of Enzymology, RCNS, Eötvös Loránd Research Network, H-1117, Budapest, Magyar tudósok krt. 2, Hungary

[6] Doctoral School of Biology and Institute of Biology, ELTE Eötvös Loránd University, H-1117 Budapest, Pázmány P. stny. 1/C, Hungary

[7] Enamine ltd., 78 Chervonotkatska St., 02094 Kyiv, Ukraine

[8] Department of Medicine I, Institute of Cancer Research, Comprehensive Cancer Center, Medical University of Vienna, Vienna, Austria

*Correspondence: barbara.zdrazil@univie.ac.at; gerard@lacdr.leidenuniv.nl

[$] Shared first authorship

Table S1: Pairwise sequence identity/similarity of hepatic OATPs calculated via Emboss NEEDLE algorithm.

OATP pairs	Sequence identity	Sequence similarity
OATP1B1-OATP1B3	552/703 (78.5%)	612/703 (87.1%)
OATP1B1-OATP2B1	229/759 (30.2%)	359/759 (47.3%)
OATP1B3-OATP2B1	226/753 (30.0%)	364/753 (48.3%)

Table S2: Range of physicochemical properties for known (published) OATP1B1, OATP1B3, and OATP2B1 ligands.

Molecular descriptor	OATP1B1	OATP1B3	OATP2B1
SlogP	-0.3 - 8.1	-0.3 - 8.7	-1.1 - 8.9
TPSA	0 - 275.6	0 - 297.1	9.2 - 241.9
SMR	37.6 - 245.1	33.1 - 292.1	40.4 - 216
# Rotatable bonds	0 - 20	0 - 20	1 - 17
AMW	136.2 - 936.9	128.6 - 1,093.3	144.2 - 794

Table S3: Compounds belonging to A) category "G1" (potentially OATP1B1 selective compounds), B) category "G2" (potentially OATP1B3 selective compounds), and C) category "G3" class (potentially OATP2B1 selective compounds), respectively. Compound identfiers, InChiKeys, ZINC IDs, and canonical smiles of the respective compounds are provided in the tables.

A)

ID	InChiKey	ZINC ID	Canonical_Smiles
A2	ACYGHNNXSSYSJV-UHFFFAOYSA-N	Z18183836	O=C(COC(=O)CN1C(=O)NC2(CCc3ccccc23)C1=O)Nc4sc5CCCCCc5c4C#N
C2	ASLLQSGMEYZPAP-UHFFFAOYSA-N	Z30403010	Cc1cc(C)n2nc(SCc3ccccc3C(=O)NCCc4ccc5OCCOc5c4)nc2n1
F5	CQKPHKQOVZZOGR-UHFFFAOYSA-N	Z27739956	Cc1cc(NS(=O)(=O)c2ccc(NC(=O)Cc3coc4c(C)c(C)ccc34)cc2)nc(C)n1
D6	CVXSCHJOJGBPAZ-UHFFFAOYSA-N	Z73253870	O=C(CN1C(=O)NC2(CCCc3ccccc23)C1=O)Nc4nnc(s4)C56CC7CC(CC(C7)C5)C6
E2	FYUUHSABTUNTET-UHFFFAOYSA-N	Z15580277	COc1ccc(cc1)C2(NC(=O)N(CC(=O)c3ccc4OCOc4c3)C2=O)c5ccc(OC)cc5
D3	JAADGCNRRNIBEX-UHFFFAOYSA-N	Z73356179	O=C(CCN1C(=O)NC2(CCCC2)C1=O)Nc3nc(cs3)c4ccc5CCCc5c4
D2	LIRJXXNACOAWNR-UHFFFAOYSA-N	Z65993343	O=C(CN1C(=O)NC2(CCCc3sccc23)C1=O)N4C(COc5ccccc45)c6ccccc6
F6	RPQZQKIWSPLTPO-UHFFFAOYSA-N	Z236281476	O=C(NCc1ccc(nc1)N2CCOCC2)c3ccccc3CSc4nc5ccccc5[nH]4

G5	UVDJCMKKFZWLPW-UHFFFAOYSA-N	Z16202316	COc1cc2c(oc3ccccc23)cc1NC(=O)CN4C(=O)NC(Cc5c[nH]c6ccccc56)C4=O
E3	DUPDCPDVSSYCDR-UHFFFAOYSA-N	Z239083200	CN1C(=O)NC(=O)c2c1nc(N\N=C/3\C(=O)Nc4ccccc34)n2CCCc5ccccc5
H6	RGHNYOWRPSUSBI-UHFFFAOYSA-N	Z31197078	CC(=O)Nc1ccc(cc1)S(=O)(=O)Nc2nc3ccccc3nc2NCc4ccccc4
A6	UZLHHAKFFPLYKS-UHFFFAOYSA-N	Z16202993	O=C(CN1C(=O)NC(Cc2c[nH]c3ccccc23)C1=O)Nc4ccc(OCc5ccccc5)cc4
A3	YFCSBGKVYQDUOD-UHFFFAOYSA-N	Z24547625	Cc1occc1c2nnc(SCC(=O)c3ccc4OCC(=O)Nc4c3)n2Cc5ccccc5
H5	ZZTAEHWHAKRXJL-UHFFFAOYSA-N	Z14227232	COC(=O)c1c2CCCCCc2sc1NC(=O)CN3C(=O)NC4(CCc5ccccc5C4)C3=O

B)

ID	InchiKey	ZINC ID	Canonical_Smiles
G2	CMPNHCNFGMIIGN-UHFFFAOYSA-N	Z96028440	O=C(Nc1ccccc1)C2CCN(CC2)C(=O)c3cc(nc4ccccc34)c5ccccc5
H4	DBSOPUYZJVXKIF-UHFFFAOYSA-N	Z16276741	Cc1cc(C)c(C(=O)CSC2=Nc3ccccc3C(=O)N2Cc4ccc(F)cc4)c(C)c1
G6	FQRDZCVYXMKEEP-NKFKGCMQSA-N	Z57055611	Cc1ccc(N2C(=O)\C(=C/Nc3nc4ccccc4nc3N5CCCC5)\c6ccccc6C2=O)c(C)c1

D5	GCWGZTMLJPMEGO-UHFFFAOYSA-N	Z14674222	O=C(COC(=O)C1=NN(C(=O)c2ccccc12)c3ccccc3)N(C4CCCCC4)C5CCCCC5
C7	GUYNZYNXRHOXRJ-BUHFOSPRSA-N	Z95128013	OC(=O)c1cc(\C=C\c2c(nc3ccccn23)c4ccccc4)nc5ccccc15
C6	HKGQAMMYWKWYIA-UHFFFAOYSA-N	Z131405274	CC(C)c1cc(C(=O)N2CCC(CC2)N3C(=O)Nc4ccccc34)c5cnn(C(C)C)c5n1
A4	HSSTUYCFXDZSCI-UHFFFAOYSA-N	Z48951465	Fc1ccccc1c2cc(c3ccccc3)c4c5CCCCc5sc4n2
A5	IEAVPOZMUKMNRR-UHFFFAOYSA-N	Z56811116	Fc1ccc(cc1)C2CC(=NN2c3nc(c4ccccc4)c5cc(Cl)ccc5n3)c6cccs6
C3	JYVNEQHJQPFBCU-UHFFFAOYSA-N	Z71034062	CCc1ccccc1NC(=O)CN2CCN(CC2)C(=O)c3cc(nc4ccccc34)c5ccncc5
B4	MDQNZPPILBWWNA-UHFFFAOYSA-N	Z243555676	[O-][N+](=O)c1ccccc1CC(=O)NCC2CC3c4ccccc4C2c5ccccc35
G4	AOYRYPNIZRIOPC-UHFFFAOYSA-N	Z25352843	CCC(C)c1ccc(cc1)N2C(=Nc3ccccc3C2=O)SCC(=O)N4C(C)Cc5ccccc45
A7	DZFZVEBCNZKUFG-UHFFFAOYSA-N	Z850224826	CC(C)(C)c1ncc(NC(=O)c2ccccc2c3oc(cn3)c4ccccc4)cn1
B2	OXUDHPYPJCMYRZ-UHFFFAOYSA-N	Z21573978	Cc1ccc(NC(=O)C(Sc2nnc3CCCCCn23)c4ccccc4)cc1Cl
G3	RTIWKGYJDWIQBH-UHFFFAOYSA-N	Z224406276	Fc1cccc(F)c1c2oc(CCC(=O)N3CCN(CC3)S(=O)(=O)c4ccccc4F)nc2

C5	ZTHGCANZNVAXSF-UHFFFAOYSA-N	Z56879983	Cc1cccc2nc(CSC3=Nc4sc5CCCCc5c4C(=O)N3Cc6ccccc6)cn12

C)

ID	InchiKey	ZINC ID	Canonical_Smiles
B7	CJDVVSPVLWQFME-UHFFFAOYSA-N	Z16710877	CSCCC(NC(=O)c1occc1)C(=O)OCC2=CC(=O)Oc3cc(O)ccc23
B5	DYZNNEVLMFDZJK-UHFFFAOYSA-N	Z30166414	O=C(NCc1ccccn1)c2ccc3C(=O)c4ccccc4S(=O)(=O)c3c2
F4	FJOQXHWRFCELRJ-UHFFFAOYSA-N	Z360489130	Cc1ccc(OCc2oc(SCC(=O)N3CCN(CC3)C(=O)Nc4ccccc4)nn2)cc1
E4	IQVYNHKDDMOEQL-UHFFFAOYSA-N	Z223702056	CC1CCN(CC1)c2nnc(SCC(=O)NCC3COc4ccccc4O3)n2C5CC5
D7	JAIMDCXKLMUECP-UHFFFAOYSA-N	Z115131598	[O-][N+](=O)c1ccc(OCC(=O)Nc2c(oc3ccccc23)C(=O)N4CCOCC4)cc1
F2	KHXILQTWNCIXFU-UHFFFAOYSA-N	Z18319532	O=C(COc1ccccc1C(=O)Nc2ccccc2)N3CCN(CC3)C(=O)c4occc4
B6	KWJPWLKIEWPVES-UHFFFAOYSA-N	Z15929440	FC(F)(F)c1ccc2c(c1)nc(SCC(=O)c3ccc[nH]3)n2Cc4ccccc4
F3	MBYCVSKUVBLZPN-UHFFFAOYSA-N	Z109476484	O=C(Nc1ccccc1)N2CCCC(C2)C(=O)N3CCN(Cc4nc5ccccc5s4)CC3
D4	MOAJPIHKTNZQRY-UHFFFAOYSA-N	Z92390611	CC(OC(=O)c1cc(Cl)c2OCCCOc2c1)C(=O)c3ccc4OCC(=O)Nc4c3

H3	YXFIRCJCYXQNDX-UHFFFAOYSA-N	Z20105105	CC(OC(=O)C1CCN(CC1)S(=O)(=O)c2ccc3OCCOc3c2)C(=O)Nc4cccc(c4)C#N
B3	GBLMNDDLVKBJTR-UHFFFAOYSA-N	Z33531613	Fc1ccccc1S(=O)(=O)N2CCN(CC2)C(=O)CNC(=O)CC34CC5CC(CC(C5)C3)C4
H2	IUNIUZZGPFECNN-OUKQBFOZSA-N	Z109445254	O=C(\C=C\c1cn(Cc2ccccc2)nn1)N3CCCC(C3)c4nc5ccccc5s4
E5	JINQIDKLFORJPN-UHFFFAOYSA-N	Z227825644	Fc1cc(F)cc(c1)C(=O)NCc2cn(Cc3ccccc3)nc2c4ccccc4
C4	JSMFOXIVOLGYRG-UHFFFAOYSA-N	Z151288042	Cc1cccn2cc(COC(=O)c3ccc4C(=O)N(Cc5occc5)C(=O)c4c3)nc12
E6	PEKYVRQFLPTKKN-UHFFFAOYSA-N	Z24103984	CCn1cnc2N(Cc3ccccc3)C(=O)N(CC(=O)NC4CCCC(C)C4C)C(=O)c12

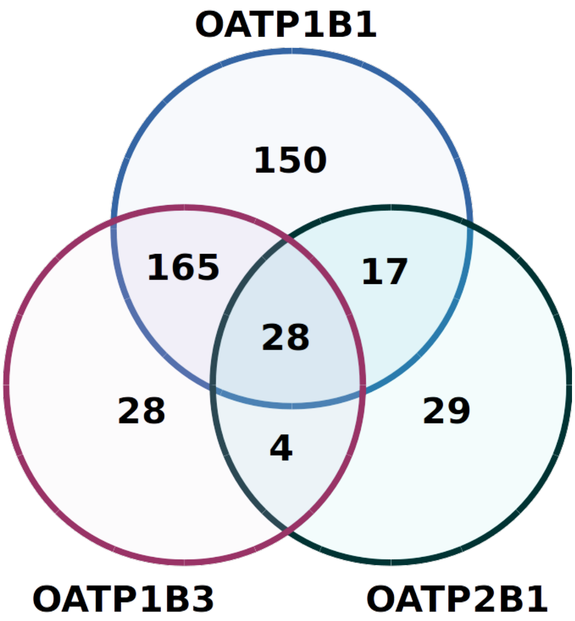

Figure S1. Venn diagram showing the overlap of active compounds for OATP1B1, OATP1B3, and OATP2B1.

A2

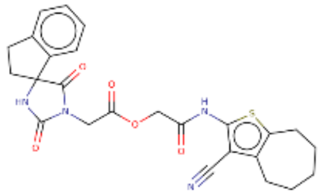

A3

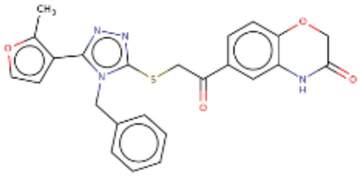

A4

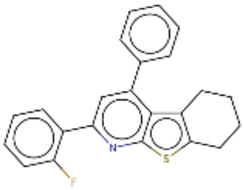

A5

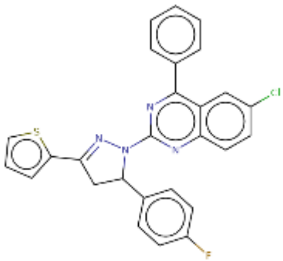

A6

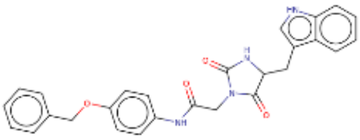

A7

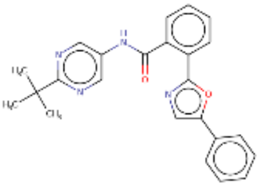

B2

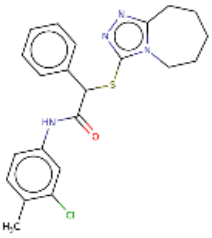

B3

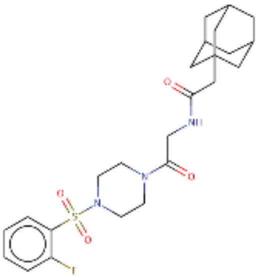

B4

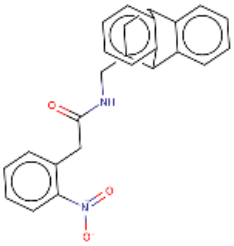

B5

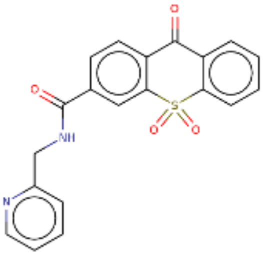

B6

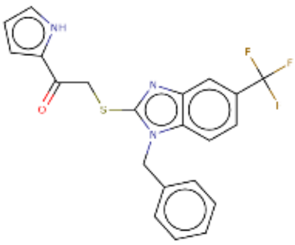

B7

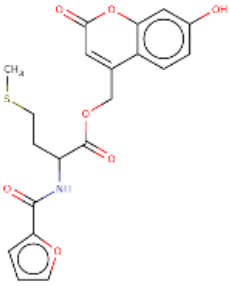

C2

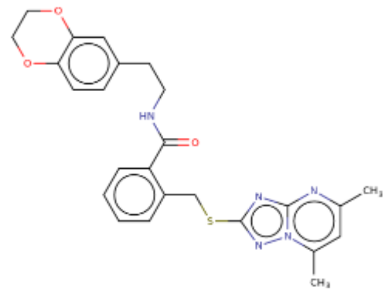

C3

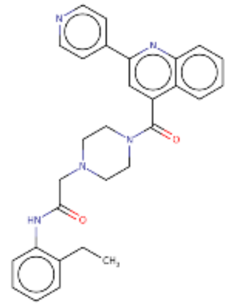

C4

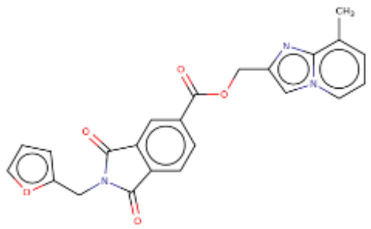

C5

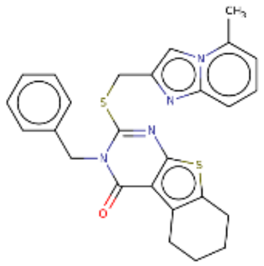

C6

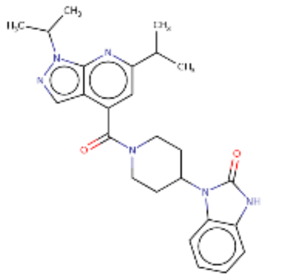

C7

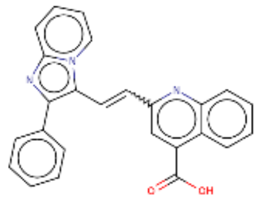

D2

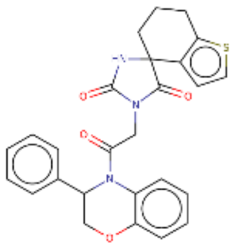

D3

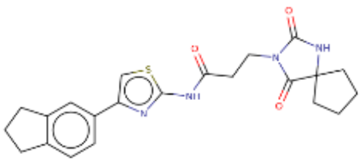

D4

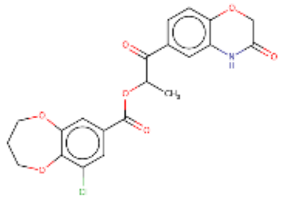

D5

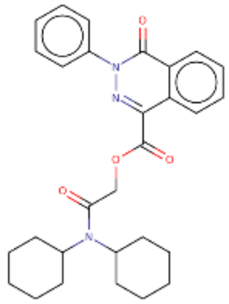

D6

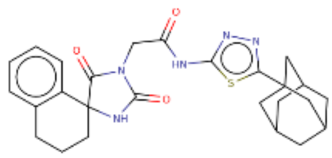

D7

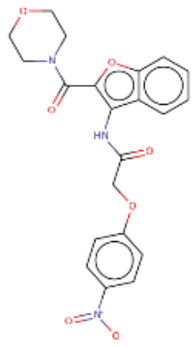

E2

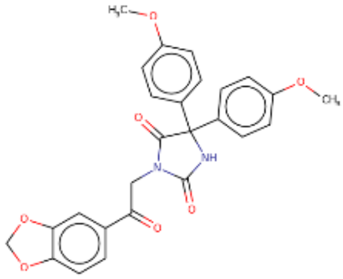

E3

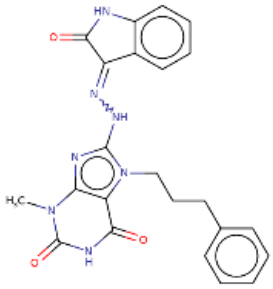

E4

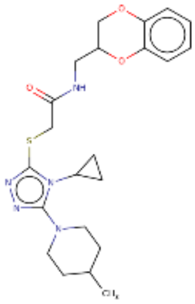

E5

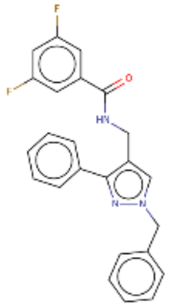

E6

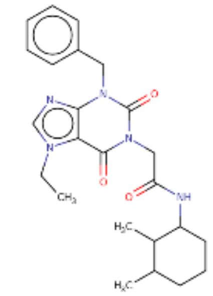

F2

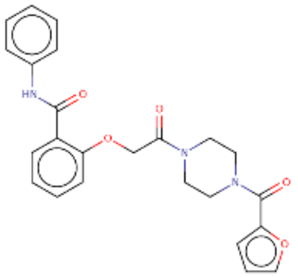

F3

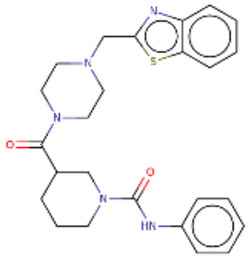

F4

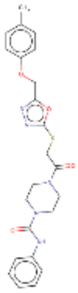

F5

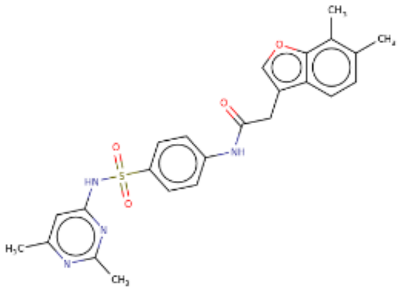

F6

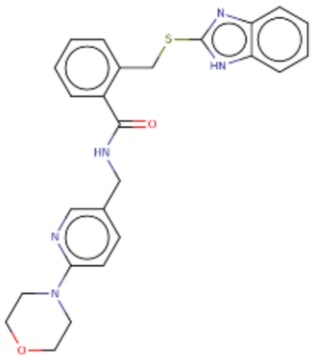

G2

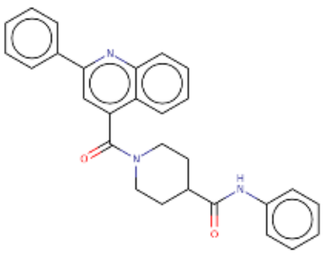

G3

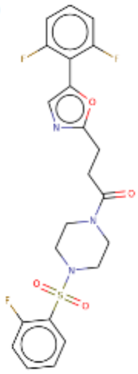

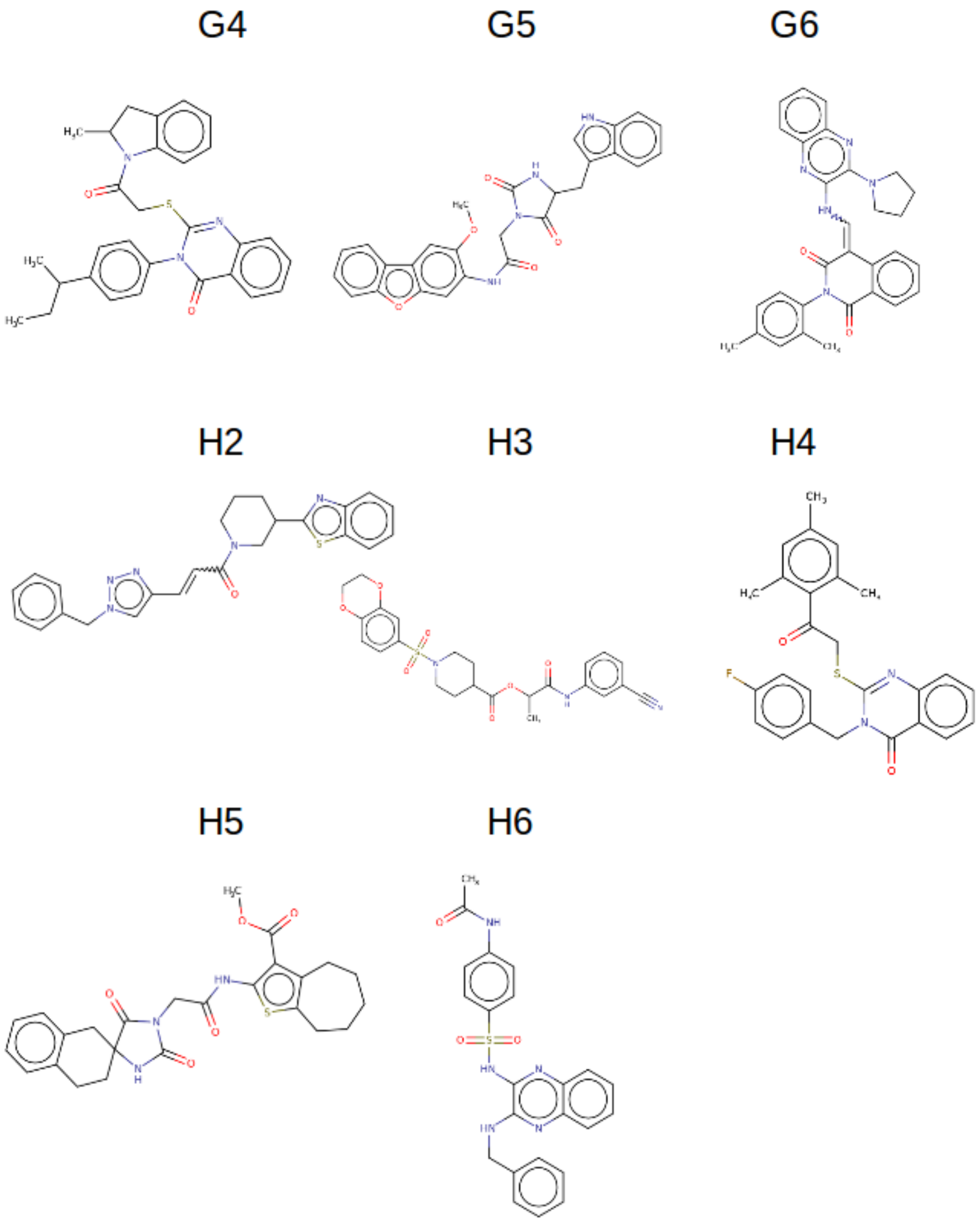

Figure S2: Chemical structures of the compound set (n=44) prioritized by ML-based virtual screening.

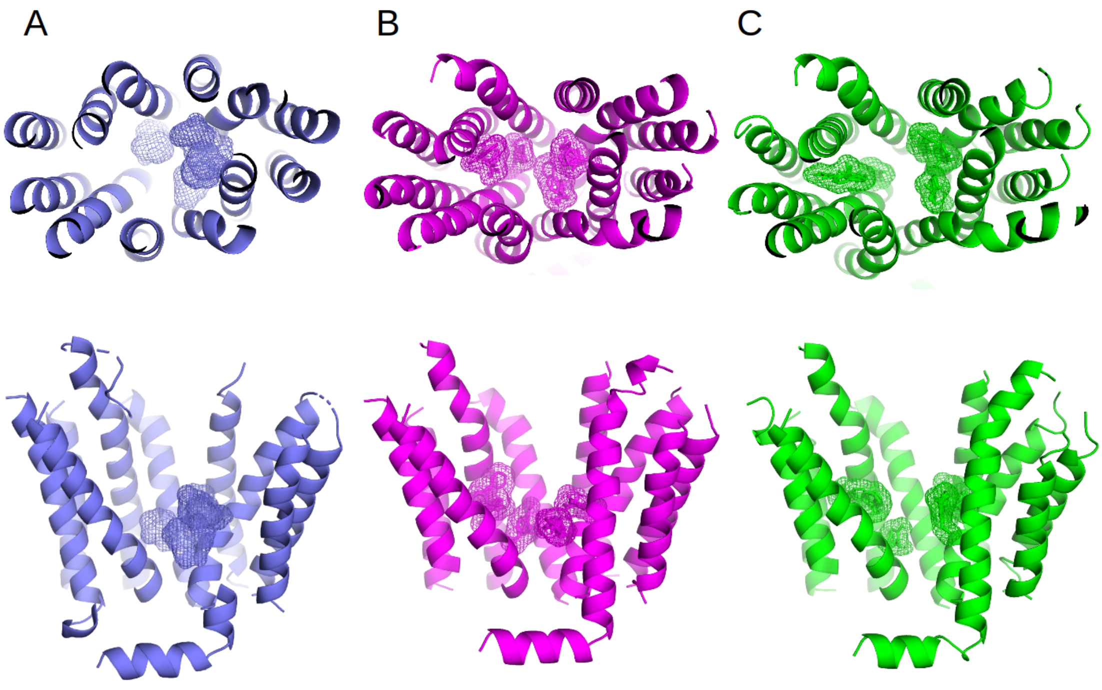

Figure S3: Predicted binding sites for (A) OATP1B1, (B) OATP1B3, and (C) OATP2B1, respectively.

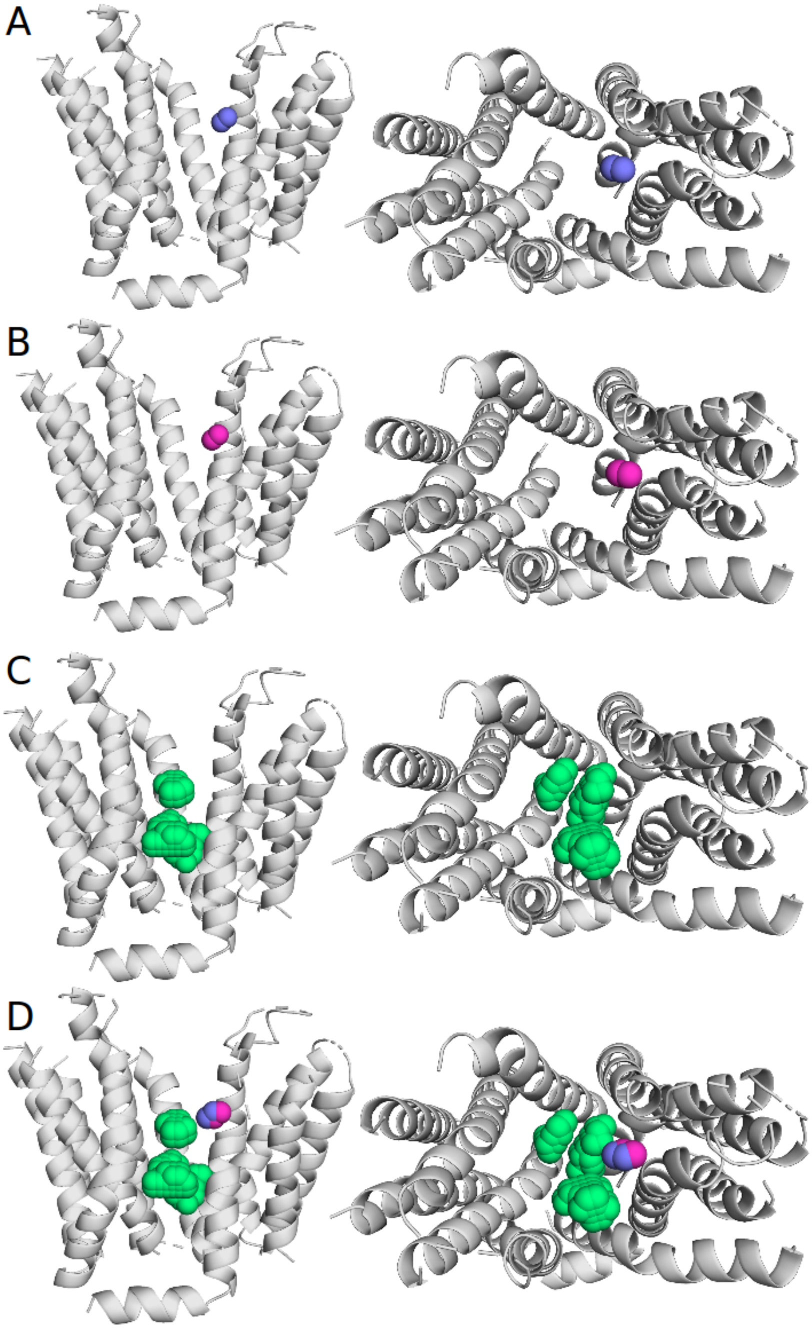

Figure S4: Volumetric map showing the distribution of aromatic residues in the binding site of (A) OATP1B1 (blue coloring), (B) OATP1B3 (magenta coloring), (C) OATP2B1 (green coloring), and (D) superimposed transporters.

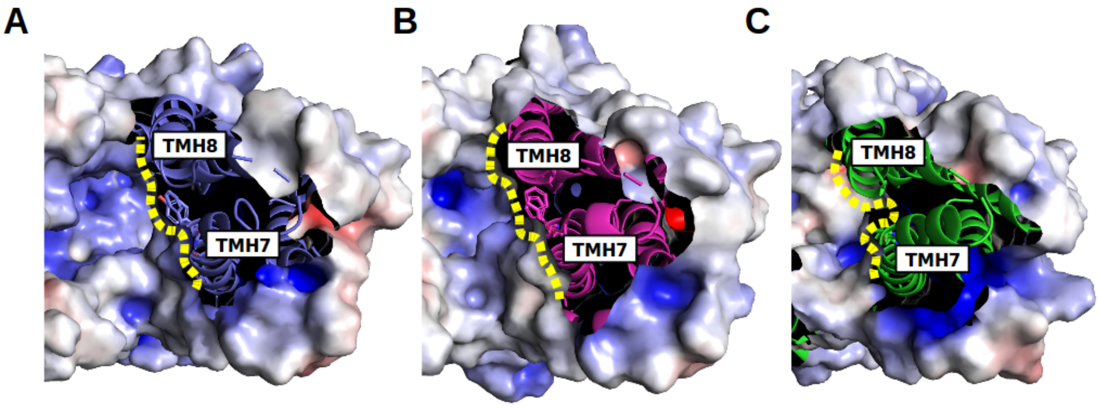

Figure S5: Electrostatic potential mapped in the (A) OATP1B1, (B) OATP1B3, and (B) OATP2B1 inner cavities (top view). Substitution of two aromatic residues in OATP1B1/OATP1B3 at position 352 (387 in OATP2B1) and 356 (391 in OATP2B1) to alanine in OATP2B1 leads to an increase of the bulk at the TMH7/TMH8 interface. Red color indicates negative electric potential, whereas blue indicates positive electric potential.

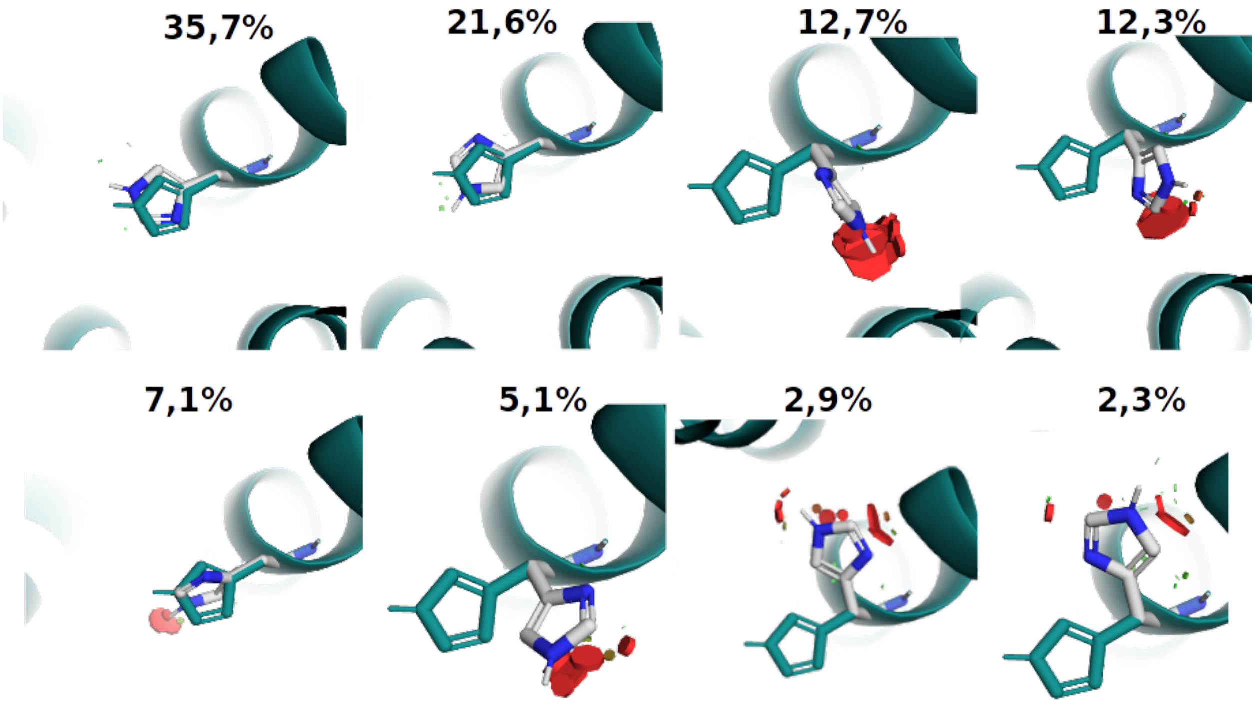

Figure S6: Possible rotamers for HIS579 in OATP2B1. Red regions show steric clashes with the surrounding residues. Percentage values indicate the occupancy of a certain rotamer. Rotamer analysis shows that HIS579 is pointing towards the central cavity of the OATP2B1 binding site, thus having an impact on pocket geometry.

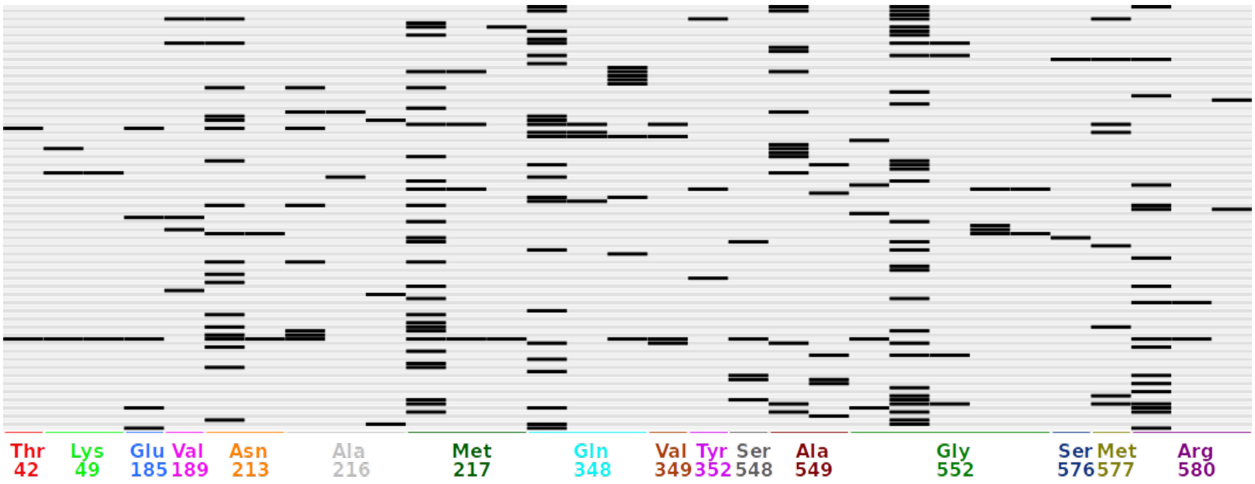

Figure S7: Protein-ligand interaction fingerprints for newly identified OATP1B1 inhibitors. Each residue presented here is labeled by a different color for better navigation in the PLIF diagram. Each row in the PLIF diagram (y-axis) corresponds to a single ligand (out of 44 ligands measured in this study, if available, multiple ligand poses were considered in the PLIF analysis). The x-axis corresponds to residues with single or multiple distinct non-bonded interactions. Distinct interaction types in MOE include the following: H-donor (cut-off 0.5-1.5 [kcal/mol]), H-acceptor (cut-off 0.5-1.5 [kcal/mol]), ionic attraction (cut-off 0.5-3.5 [kcal/mol]), metal ligation (cut-off 0.5-3.5[kcal/mol]), and arene attraction (cut-off 0.5-1.0 [kcal/mol]).

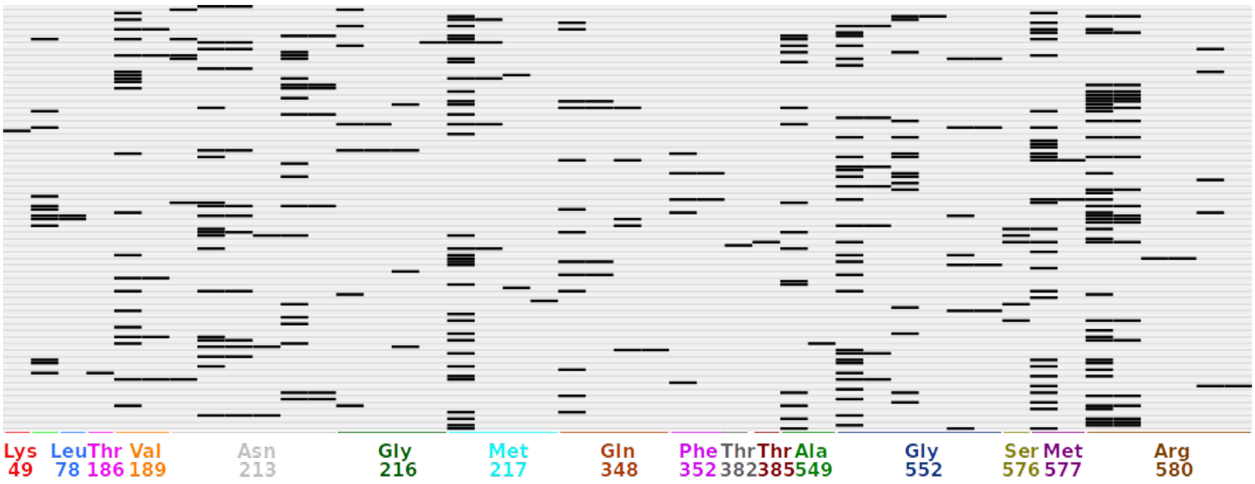

Figure S8: Protein-ligand interaction fingerprints for newly identified OATP1B3 inhibitors. Each residue presented here is labeled by a different color for better navigation in the PLIF diagram. Each row in the PLIF diagram (y-axis) corresponds to a single ligand (out of 44 ligands measured in this study, if available, multiple ligand poses were considered in the PLIF analysis). The x-axis corresponds to residues with single or multiple distinct non-bonded interactions. Distinct interaction types in MOE include the following: H-donor (cut-off 0.5-1.5 [kcal/mol]), H-acceptor (cut-off 0.5-1.5 [kcal/mol]), ionic attraction (cut-off 0.5-3.5 [kcal/mol]), metal ligation (cut-off 0.5-3.5[kcal/mol]), and arene attraction (cut-off 0.5-1.0 [kcal/mol]).

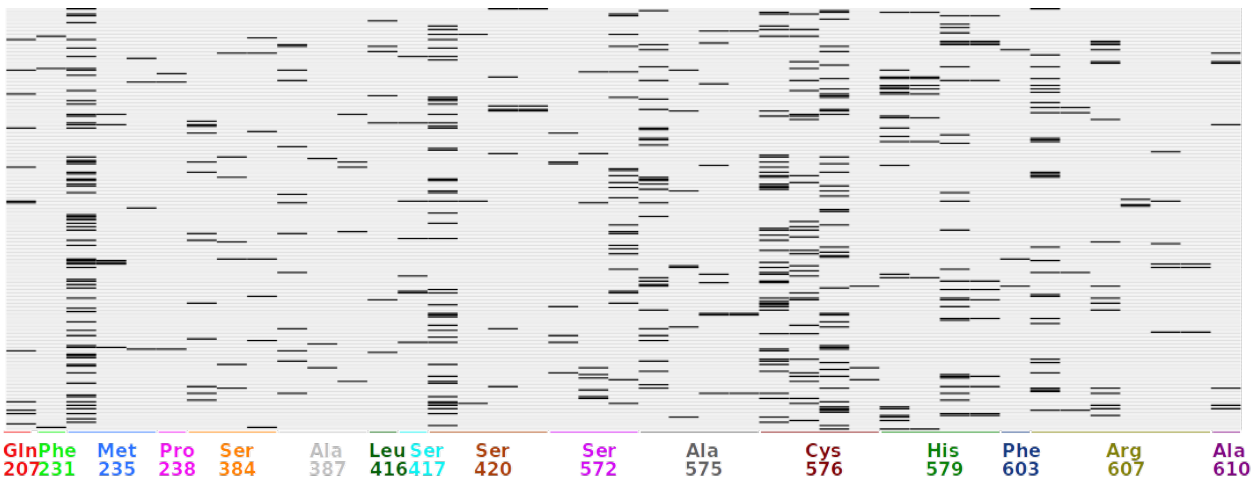

Figure S9: Protein-ligand interaction fingerprints for newly identified OATP2B1 inhibitors. Each residue presented here is labeled by a different color for better navigation in the PLIF diagram. Each row in the PLIF diagram (y-axis) corresponds to a single ligand (out of 44 ligands measured in this study, if available, multiple ligand poses were considered in the PLIF analysis). The x-axis corresponds to residues with single or multiple distinct non-bonded interactions. Distinct interaction types in MOE include the following: H-donor (cut-off 0.5-1.5 [kcal/mol]), H-acceptor (cut-off 0.5-1.5 [kcal/mol]), ionic attraction (cut-off 0.5-3.5 [kcal/mol]), metal ligation (cut-off 0.5-3.5[kcal/mol]), and arene attraction (cut-off 0.5-1.0 [kcal/mol]).

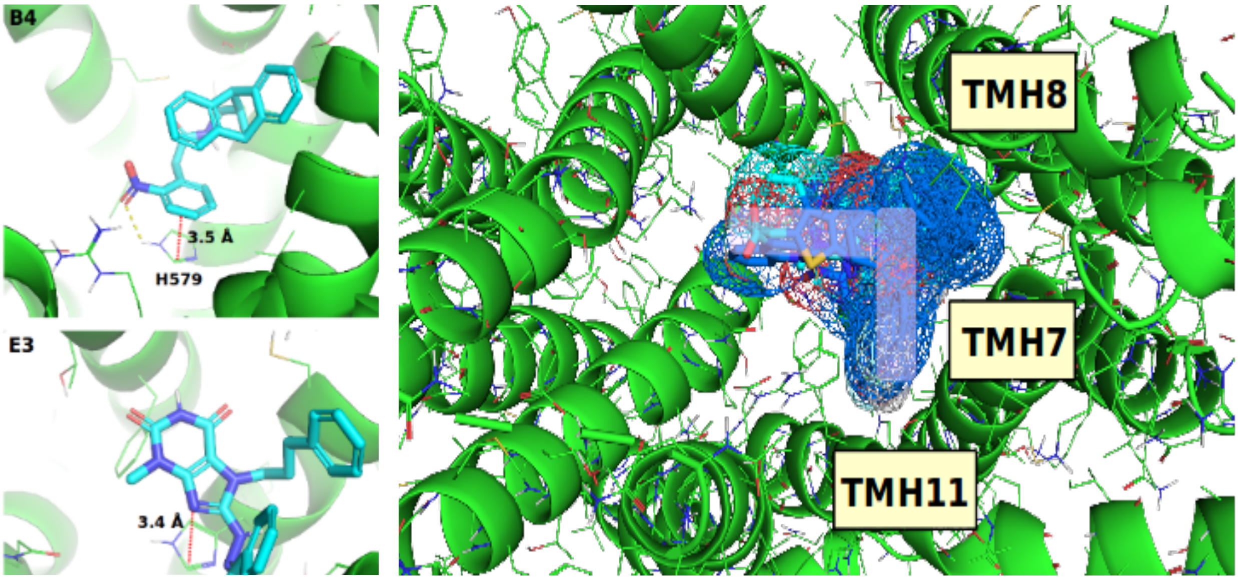

Figure S10: Docking poses of OATP2B1 inhibitors (compounds B4, C7, E3, E5, H5, and G4). **Left panel:** Compounds B4 and E3 poses in OATP2B1 (pi-pi interaction between the ligand and HIS579 are indicated by the red dashed line). **Right panel:** Representative poses for B4, C7, E3, E5, H5, and G4 compounds show the 'L-shaped' binding mode. The poses shown here represent the most populated poses per compound identified by hierarchical pose clustering.

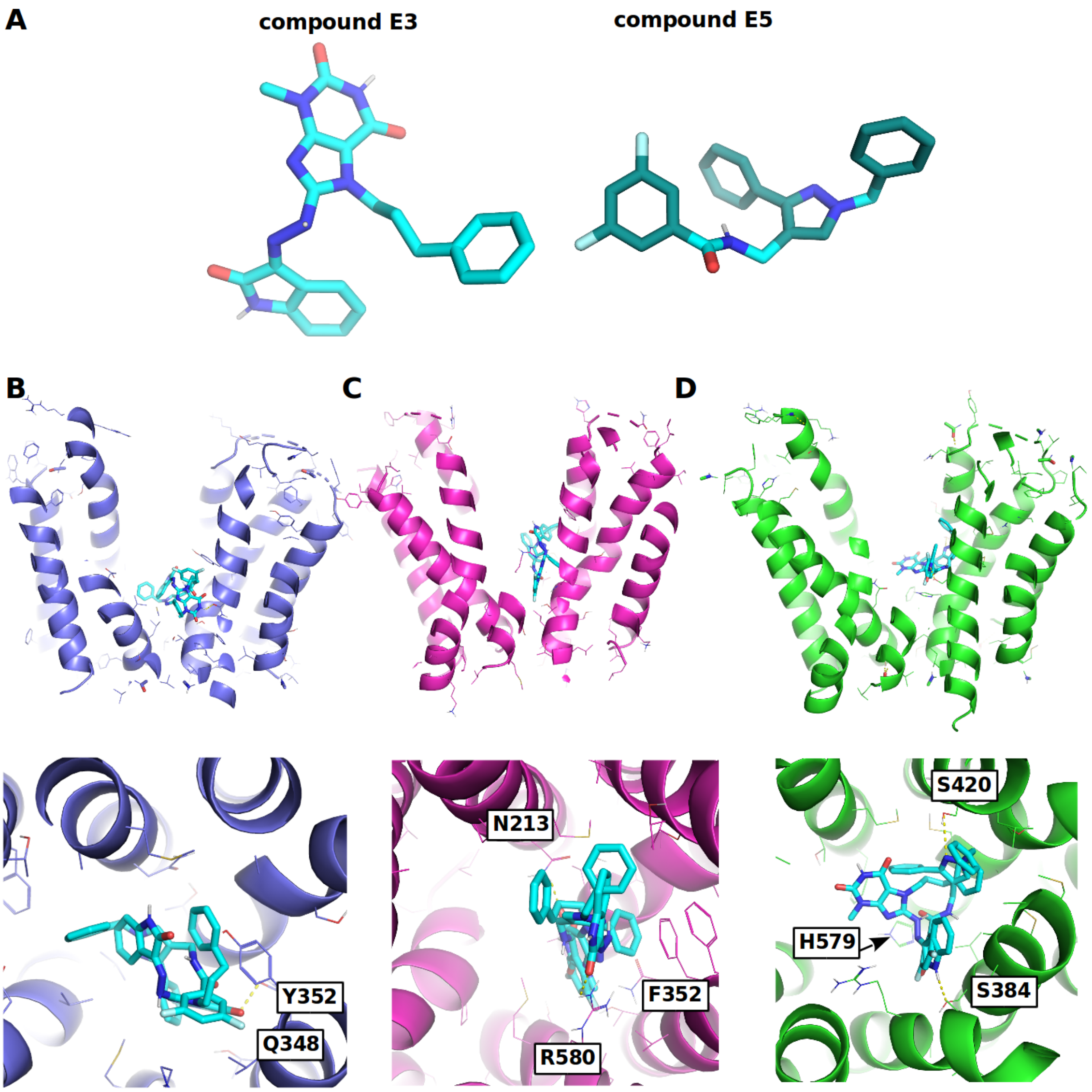

Figure S11: (A) 3D structures of compounds E3 and E5, respectively. Docking poses of compounds E3 and E5 compound in (A) OATP1B1, (B) OATP1B3, and (C) OATP2B1 transporter, respectively.

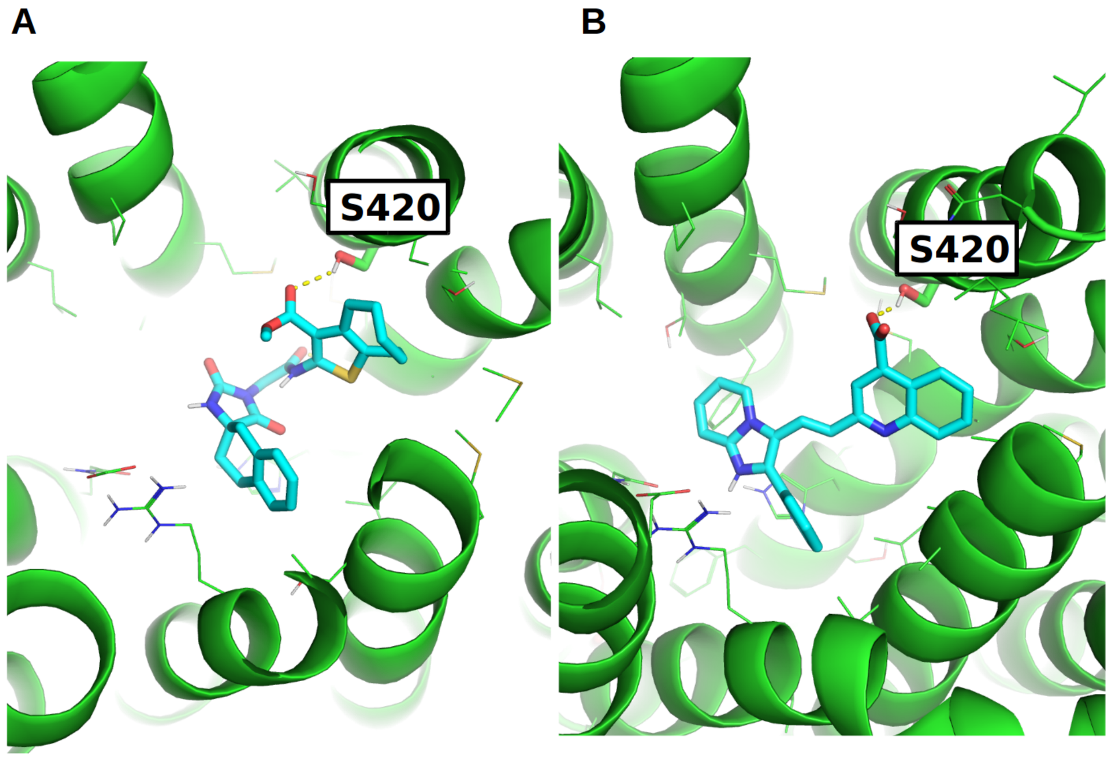

Figure S12: Docking poses for (A) compound H5 and (B) compound C7 in OATP2B1 showing hydrogen bond interaction with SER420 (a non-conserved residue across the three hepatic OATPs).
